# miR-58 family and TGF-β pathways regulate each other in *Caenorhabditis elegans*

**DOI:** 10.1093/nar/gkv923

**Published:** 2015-09-22

**Authors:** María Pilar de Lucas, Alberto G. Sáez, Encarnación Lozano

**Affiliations:** Unidad Funcional de Investigación de Enfermedades Crónicas, Instituto de Salud Carlos III, 28220, Majadahonda, Madrid, Spain

## Abstract

Despite the fact that microRNAs (miRNAs) modulate the expression of around 60% of protein-coding genes, it is often hard to elucidate their precise role and target genes. Studying miRNA families as opposed to single miRNAs alone increases our chances of observing not only mutant phenotypes but also changes in the expression of target genes. Here we ask whether the TGF-β signalling pathways, which control many animal processes, might be modulated by miRNAs in *Caenorhabditis elegans*. Using a mutant for four members of the *mir-58* family, we show that both TGF-β Sma/Mab (controlling body size) and TGF-β Dauer (regulating dauer, a stress-resistant larval stage) are upregulated. Thus, *mir-58* family directly inhibits the expression of *dbl-1* (ligand), *daf-1*, *daf-4* and *sma-6* (receptors) of TGF-β pathways. Epistasis experiments reveal that whereas the small body phenotype of the *mir-58* family mutant must invoke unknown targets independent from TGF-β Sma/Mab, its dauer defectiveness can be rescued by DAF-1 depletion. Additionally, we found a negative feedback loop between TGF-β Sma/Mab and *mir-58* and the related *mir-80*. Our results suggest that the interaction between *mir-58* family and TGF-β genes is key on decisions about animal growth and stress resistance in *C. elegans* and perhaps other organisms.

## INTRODUCTION

Although around 60% of protein-coding genes are estimated to be under the control of microRNAs (or miRNAs) and despite the general assumption that miRNAs are able to co-ordinate the expression of multiple mRNAs, the path to elucidate miRNA functions and/or interactions with target genes remains a challenge (1–3). For instance, in *Caenorhabditis elegans*, only 10 out of 434 mature miRNAs have been experimentally validated as regulators of particular target genes (miRecords April 2013; miRBase Release 21). This slow progress is caused, in part, by the fact that only a small minority of miRNAs shows an obvious phenotype when mutated (4). Hence, one way forward may be to study strains with loss-of-function mutations at various redundant miRNAs, as opposed to mutants that only affect single miRNAs. This approach not only increases our chances of observing phenotypic anomalies that we can relate to other mutants, but also of identifying target genes by observing their altered expression patterns.

In this way, Alvarez-Saavedra & Horvitz identified three miRNA-family mutants of *C. elegans* with obvious abnormalities that were mostly absent in their respective single miRNA mutants (5). One of the aberrant family strains was a quadruple mutant for the *cel-mir-58* family, which had small body size and was dauer defective. It is known that both, body size and dauer formation in worms, depend on two analogous signalling pathways, Transforming Growth Factor (TGF)-β Sma/Mab and TGF-β Dauer, respectively (6). We here ask whether the miR-58 family could regulate one or both pathways in *C. elegans*.

TGF-β pathways are key in the proliferation and differentiation of animal cells, and their core signalling pathway is conserved across metazoa (7). Upon extracellular binding of a dimeric ligand (TGF-β) to two pairs of transmembrane receptors (type I and type II), the resulting hexameric complex becomes phosphorylated at type I receptors. Then, a cascade of intracellular phosphorylations at various transcriptions factors known as Smads is triggered, which, depending on the cellular context, induces or represses specific sets of target genes. In worms, the number of proteins and pathways involved are much smaller (e.g. five ligands, three receptors) than in mammals, which simplifies its study. The two known TGF-β pathways in *C. elegans* are the already mentioned TGF-β Sma/Mab and TGF-β Dauer.

TGF-β Sma/Mab is the best-known pathway controlling body size in *C. elegans*. Ligand DBL-1 (TGF-β), which is expressed in the ventral cord (8,9), promotes growth by its effects on hypodermis (10,11), where SMA-6 (type I) and DAF-4 (type II) receptors are present (12). Worms homozygous for null mutations at several genes of this pathway show a Sma phenotype, that is, they are dwarfed, similar in length to worms defective in the *mir-58* family (6).

TGF-β Dauer is one of the signalling cascades that regulates dauer formation. Dauer is an alternative L2-L3 larval stage that is resistant to harsh environmental conditions, such as low food, high population density, or high temperatures. When worms sense those challenges, *daf-7* (TGF-β) transcription, carried out in a pair of head sensory neurons known as ASI, is silenced. In turn, this downregulation leads to a dauer phenotype (13,14). DAF-7 receptors are DAF-1 and DAF-4 (this last one shared with TGF-β Sma/Mab), and they are broadly expressed (12). Downstream Smad include activators DAF-8 and DAF-14, and inhibitors DAF-3 and DAF-5 (15–17).

What else do we know about the *mir-58* family apart from the fact that its absence leads to a small and dauer-defective mutant? This family is made of five members, *mir-58* (chromosome IV), *mir-80* (III), *mir-81* and *mir-82* (approximately 4 kb apart from each other in chromosome X), and *mir-1834*, although this last one has not been fully validated as a functional miRNA (chromosome IV; >3 Mb apart from *mir-58*) (18). The strain family mutant, MT15563, referred also as *mir-58f(-)* hereafter, holds three deletions covering the first four miRNAs. *mir-58f(-)* was described as sluggish, small, and egg-laying and dauer defective (5). In contrast, single or double *mir-58*-*family* deletions do not result in obvious developmental defects. In consequence, *mir-58(-)* apparently develops normal and only shows a 20% shorter life span (19), which is surprising considering that miR-58 is the miRNA with the highest (20), or one of the highest levels of expression (21), at every developmental stage and across multiple tissues, with the significant exception of the nervous system (22). A lower, but still broad expression has been described for miR-80 and less so for miR-82 (20). However, these two are found in neurons, and the later is only expressed from L4 onwards. In contrast to the previous three miRNAs, miR-81 has only been weakly detected in head neurons from L1 (22,23). These tissue-specificity patterns of expression suggest that cel-miR-58 members could have redundant as well as divergent functions.

There are *mir-58* orthologs in other invertebrates, like Drosophila, where it is known as *bantam* (24,25). According to some reports several human *mir-58* orthologs also exist, although this is not firmly established (21,26,27). Interestingly, *bantam* is among the few mutated miRNAs with an obvious phenotype in Drosophila, and like in nematodes, those fruitflies are small (28,29). A number of biochemical pathways have been related to *bantam*, both upstream and downstream of it (30,31). Drosophila's DPP/TGF-β is known to be one of the pathways upstream of *bantam* (32–34).

In this work, we primarily focus on the relationship between the *mir-58* family and TGF-β, Sma/Mab and Dauer, in *C. elegans*. We find that various genes from both TGF-β pathways are controlled by the *mir-58* family. We also find a positive regulation of *mir-58* transcription by TGF-β.

## MATERIALS AND METHODS

### Strains and culture conditions

Wild-type *C. elegans* N2 strain (Bristol) and the following mutant strains were obtained from Caenorhabditis Genetics Centre (CGC): BW1940 *ctIs40 X* [ZC421*(dbl-1(+))* + pTG96(*sur-5::gfp*)], CB1370 *daf-2(e1370) III*, DR63 *daf-4(m63) III*, DR609 *daf-1(m213) IV*, LT186 *sma-6(wk7) II*, MT13949 *mir-80(nDf53) III*, MT13954 *mir-81&mir-82(nDf54) X*, MT15024 *mir-58(n4640) IV*, MT15563 *mir-80(nDf53) III; mir-58(n4640) IV; mir-81&mir-82(nDf54) X*, NU3 *dbl-1(nk3*) *V*, RB1739 *sma-10(ok2224) IV*, and RB2589 *daf-3(ok3610) X*. DR2490 *mIs27 [P_daf-8_::daf-8::gfp, rol-6(su1006)]*, EUB0032 *P_mir-58_::gfp* and *pwIs922* [*P_vha-6_::daf-4::gfp*] were kindly provided by Drs D. Riddle, M. Isik and R. Padgett, respectively. Strains were cultured on agar plates seeded with *Escherichia coli* OP50 and incubated at 20°C according to standard procedures (35), except for CB1370, DR63 and DR609 that were cultured at 15ºC. MT13949, MT13954, MT15024 and RB1739 were outcrossed to N2 for four to six generations before any test was performed. Moreover, we generated combined mutants of the above strains, as well as transgenic animals that we also crossed with those mutants (see below and Supplementary Table S1). The presence of each correspondent mutation was confirmed by PCR and electrophoresis, and if necessary, by DNA sequencing (primers in Supplementary Table S2).

### Epistasis analyses

To study body size, synchronized worms grown individually on 5 cm NGM Petri dishes were measured at 24 h intervals since around 100 h post-hatching until they reached plateau sizes. Final lengths were calculated on averages of two to three consecutive days, when the worms had reached a plateau size. Images were captured using a video camera (KY-F550, JVC Professional Europe Ltd.) attached to a dissecting microscope (x50, Leica MZ7.5), and images were analysed with ImageJ software (1.46r; National Institutes of Health). A minimum of two independent experiments was carried out for each strain and treatment, and a range of 20–139 worms was measured per strain.

For epistatic dauer assays, we made worms deficient in both *daf-1* and the *mir-58* family by repeatedly backcrossing DR609 males to MT15563 hermaphrodites until we obtained a compound mutant from both. We followed the same protocol to create *daf-2(e1370);mir-58f(-)* mutants. Dauer assays were performed as follows: young adult hermaphrodites of N2, MT15563, DR609, CB1370, *daf-1(m213);mir-58f(-)* and *daf-2(e1370);mir-58f(-)* mutants were let to lay eggs for 4 h at 20ºC. After removing the hermaphrodites, NGM plates were incubated at 20ºC or 25.5ºC up to 3 days and dauers were scored. Animals were anesthetized with 25 mM NaN_3_ in M9 and pictured with a confocal microscope (Leica DMI 6000) under 63x objective (for pharynx images) and Zeiss Axio Imager.A1 microscope with 100x objective under Nomarski conditions (for alae).

Statistical pairwise comparisons were run on R version 2.15.1. We first checked whether each data set had a normal distribution, by using the Shapiro-Wilk normality test. In case normality was rejected, data would be log-transformed. Then, pairwise comparisons were performed with a Welch Two Sample *t*-test. The same procedure was also used for obtaining *P*-values throughout this report, with the exception of the qPCRs for which we used a specific methodology (see below).

### Quantitative real-time PCR assay (qPCR)

We synchronized worms and isolated their total RNA with miRNeasy Kit (Qiagen), then used to quantify both mRNA and miRNA expression.

For quantification of mRNAs, we synthesized cDNAs with SuperScript III First-Strand Synthesis System (Invitrogen). Real-time PCR of cDNAs was run on an Applied Biosystems 7500 Fast thermocycle, using TaqMan Assays (Applied Biosystems). We tested each sample in triplicate, and analysed relative changes of transcripts by the 2–ΔΔCT method (36), using levels of *crn-1* and *cey-1* transcripts as control. *P*-values were calculated with REST software (37).

For miRNA assessment, we conducted reverse transcription and real-time quantification for each miRNA by using TaqMan MicroRNA Assays (Applied Biosystems) and the same thermocycle than above. The normalization of miRNA real-time PCRs was performed with *geNorm* (38) and *NormFinder* (39) algorithms, whereby the two most stable miRNAs across samples were miR-47 and miR-81 for TGF-β Sma/Mab genes, and miR-81 and miR-82 for the TGF-β Dauer pathway.

### Luciferase assays

We grew HeLa cells in Dulbecco's modified Eagle's medium (Lonza) with 10% FBS in 5% CO_2_ at 37ºC and seeded them at a density of 1 × 10^4^ cells per well into 96-well plates. Cells were transfected in triplicate 24/48 h later with Lipofectamine 2000 (Invitrogen), 150 ng of a 3′UTR luciferase vector (see below), and 50 nmol of test miRNA mimic (miR-58, miR-80, miR-81 and miR-1834; miRIDIAN, Dharmacon) or the standard control miRNA mimic miR-67 provided by the manufacturer. The 3′UTRs (wild-type and mutant) of *sma-6*, *dbl-1*, *daf-1*, *daf-7* and *daf-4* were cloned after the renilla reporter gene in the vector psiCHECK-2 (Promega). For obtaining the 3′UTR mutants, we mutated the second, third and fourth positions of the predicted *mir-58* family binding sites in 3′UTRs by PCR (primers in Supplementary Table S3). Dual-luciferase reporter assay were performed 48 h after transfection using Dual-Luciferease Reporter Assay System (Promega) to detect firefly and renilla luciferase activity, and luminescence was measured with an Infinite M200 TECAN luminometer (TECAN Group Ltd). Renilla luciferase activity was first normalized using the firefly luciferase activity as intraplasmid transfection reporter. Resulting values for miRNA-3′UTR co-expression were further normalized to those from the same 3′UTRs but incubated with control mimic miRNA (miR-67).

### *In vivo* 3′UTR activity assays

To test *in vivo* 3′UTR activity, we made sensor mCherry constructs with either the wild-type 3′UTR of each target gene or a mutated version as described in the luciferase assays and Supplementary Table S3. To obtain reliable mCherry quantifications when comparing the transgenic lines generated in the wild-type N2 or *mir-58f(-)* MT15563 backgrounds we did two things. First, each transgenic line generated in the N2 background were outcrossed to the MT15563 strain, in order to obtain worms in a *mir-58f(-)* background with the same arrays, or as similar as possible, than their N2 counterparts. That required backcrosses through various generations and subsequent genotype confirmation. Secondly, we included a *P_sca-1_::gfp* construct in all our arrays, so that we could use GFP levels to normalize the expression of mCherry in every worm. Thus, mCherry constructs were mixed with *P_sca-1_::gfp* (pGK10 plasmid, (40)), hygromycin resistant vector pHygroSfi (a gift from Dr J. Pérez-Martín) and a 1 kb DNA ladder (Invitrogen; see Supplementary Table S4 for concentrations of each component). For generating transgenic lines, we used standard microinjection techniques (41). Most often, to calculate reporter expression, we used two or more transgenic lines from each microinjected mixture. Animals were mounted in agar pads and pictured at specific stages under a Leica stereomicroscope (M165FC) attached to a camera (Leica DFC360FX). Pictures were analysed with ImageJ by measuring the whole-body area of worms.

To make our *mCherry::3′UTR* constructs we used a modified version of the nematode expression vector pPD95.77 (Andrew Fire collection, Addgene), having mCherry instead of GFP. We inserted different promoters and 3′UTRs depending on each experiment (Supplementary Tables S2 and S3). Thus, to make *P_sma-6_::mCherry::3′UTR_sma-6WT_* and *P_sma-6_::mCherry::3′UTR_sma-6MUT_* we amplified 3 kb of the *sma-6* 5′-flanking region (10), and also either a wild-type, or a mutated version, of *sma-6* 3′UTR (216 bp long). For *P_daf-4_::mCherry::3′UTR_daf-4_* we used 3 kb containing the *daf-4* promoter (12), and a 476 bp of the wild-type or mutated 3′UTR of *daf-4*. For *P_daf-1_::mCherry::3′UTR_daf-1_*, a 2.5 kb fragment with *daf-1* promoter (12), and 455 bp of its wild-type or mutated 3′UTR. And for *P_dbl-1_:: mCherry::3′UTR_dbl-1_* we used 2.8 kb of the 5′-flanking region of *dbl-1*, as well as 470 bp of *dbl-1* 3′UTR, wild-type or mutated.

### RNA interference

Synchronized L4 worms were fed HT115 (DE3) bacteria expressing *sma-6* or *daf-4* dsRNA on NGM plates. Adult body size of the progeny was measured (as described in ‘Epistasis analyses’) every 24 h, up to around 140 h, when they had already reached their final body size. The *sma-6* RNAi plasmid included a genomic fragment of *sma-6*, obtained by PCR with forward 5′-CAGCTGTACACGGAACTGG and reverse 5′-TCAACTTTACGCTGCGATTG oligonucleotides. This fragment was inserted into L4440 vector (Addgene) and transformed into HT115 (DE3) bacteria. The *daf-4* RNAi plasmid was purchased from Source BioScience (C05D2.1/III-2P04). Clones were cultured overnight in LB with 200 μg/ml ampicillin and 12.5 μg/ml tetracycline. We spread 120 μl of this bacterial culture onto each NGM plate supplemented with 1 mM IPTG and 25 μg/ml carbenicillin. Plates were left overnight to induce dsRNA before adding the worms. We measured at least 20 worms per strain in each of two independent experiments.

### Quantification of hyp 7 nuclei

For comparing the number of hyp 7 nuclei between N2 and MT15563, we microinjected both strains with a hypodermal marker, *P_dpy-7_::4xNLS::gfp*, together with pHygroSfi and an mCherry microinjection marker (p374, a gift from Dr A. Miranda-Vizuete (42); Supplementary Table S4). Thus, GFP expression was under the control of *dpy-7* promoter (43) and, because of its nuclear-localization sequence (NLS), it was only localized at nuclei. To make this construct, we replaced the *rpl-28* promoter of L4455 vector (Addgene) by a 616 bp fragment encompassing the *dpy-7* promoter (see Supplementary Table S2). We used a Leica stereomicroscope (M165FC) to count hyp 7 nuclei in 18 animals of each strain, which were kept at approximately 5ºC to keep them immobile during nuclei counting. We only counted hypodermal nuclei from one side of each worm, excluding dorsal and ventral nuclei.

### TGF-β Sma/Mab reporter activity

We chose *sma-6* promoter as reporter of the Sma/Mab pathway, as it is known that such pathway positively controls *sma-6* transcription (44). Our *P_sma-6_* plasmid construct was very similar to previously described *P_sma-6_::mCherry::3′UTR_sma-6WT_* (see ‘*In vivo* 3′UTR activity assays’). The only difference is that here the 3′UTR was the original *unc-54* present in pPD95.77. We did not include *3′UTR_sma-6_* to limit all expression differences to promoter control, excluding differences based on miRNAs. Transgenic worms were generated by microinjection of N2 with *P_sma-6_::mCherry::3*′*UTR_unc-54_*, together with a GFP marker (pGK10) and the other DNAs already mentioned (‘*In vivo* 3′UTR activity assays’; Supplementary Table S4). Several independent transgenic lines generated in the wild-type background were repeatedly backcrossed to MT15563, to test whether the TGF-β Sma/Mab signalling is up or down in the *mir-58* family mutant with respect to N2. As control, we also crossed the transgenic lines with either NU3 *dbl-1(nk3)* or BW1940 *dbl-1(++)*, for which Sma/Mab is known to be down- and up-regulated, respectively (8,9). Pictures and analysis of L4 worms, again referring mCherry reporter levels to GFP control expression of the extrachromosomal array, were performed as previously explained (‘*In vivo* 3′UTR activity assays’).

### TGF-β Dauer pathway reporter activity

For estimating TGF-β Dauer pathway activity in *mir-58* mutants, we used *daf-7* and *daf-8* that are known to respond positively to this pathway (45). For quantification of transcriptional activation of both genes, we performed qPCR in mixed stage populations of a variety of genetic backgrounds using specific *daf-7* and *daf-8* TaqMan probes (Applied Biosystems).

### Transcriptional regulation of *P_miR-58_*

EUB0032 *P_mir-58_::gfp* was crossed with NU3 *dbl-1(nk3)*, RB1739 *sma-10(ok2224)*, RB2589 *daf-3(ok3610)*, DR609 *daf-1(m213)* and DR63 *daf-4(m63)* to test whether the inhibition of the TGF-β pathways may regulate the activity of *P_mir-58_*. To check how the increase of Sma/Mab signalling affects *P_mir-58_* regulation, we microinjected the fosmid WRM0624CB02, which carries the *dbl-1* gene, together with other DNAs (see Supplementary Table S4 for transgene details and concentrations) into EUB0032. As a control of this last experiment, we microinjected the strain EUB0032 with the same DNA mixture except for the *dbl-1* fosmid. In all these tests we analysed GFP expression in L4 worms as previously described.

## RESULTS

Our hypothesis was that the *mir-58* family could regulate body size and dauer response through TGF-β Sma/Mab and TGF-β Dauer pathways, respectively. Then we first carried out a computational search looking for miR-58 targets related to these two pathways. Our quest in eight different databases (Microcosm targets, microrna.org, Target Scan, PICTAR, mirWip, Diana Lab, RNA22 and mirSom) rendered three putative miR-58 target genes belonging to the TGF-β Sma/Mab pathway: *dbl-1*, *daf-4* and *sma-6*. Genes *daf-1* and *daf-7* –apart from *daf-4*– all of the TGF-β Dauer pathway, also became predicted targets of miR-58. The predicted binding sites for each of these genes are summarized in Supplementary Table S5.

### mRNA levels of *dbl-1*, *sma-6*, *daf-4*, *daf-1* and *daf-7* are upregulated in the *mir-58* family mutant

**Figure 1.**
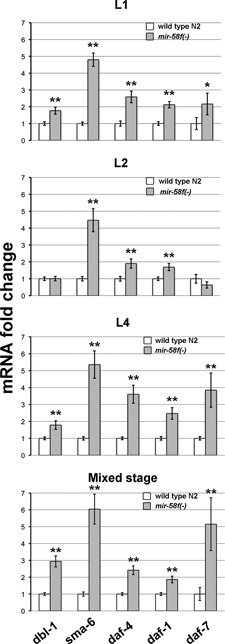
*dbl-1*, *sma-6*, *daf-4, daf-1* and *daf-7* mRNA levels are upregulated in the *mir-58* family mutant. mRNA levels of the above genes were measured in larval stages L1, L2, L4 and a mixed stage population of wild-type strain N2 and MT15563 (*mir-58f(-)*), which lacks four miRNAs of the *mir-58* family. Measurements were carried out by quantitative Real-Time PCR (qPCR) with gene-specific TaqMan probes. mRNA levels of MT15563 (grey bars) were normalized to those of N2 (white bars). Each value represents the average from four independent experiments. Error bars indicate standard deviations. Significant statistical differences between N2 and MT15563 are indicated as *(*P <* 0.01) or **(*P <* 0.001).

We performed quantitative real-time PCR (qPCR) on *mir-58f(-)*, the *C. elegans* strain missing four of the five miRNAs of the *mir-58* family (see Materials and Methods). As shown in Figure 1 (mixed stage, bottom panel), the expression of *dbl-1*, *daf-4*, *daf-1, daf-7* and most notably *sma-6* (sixfold), was increased in *mir-58f(-)* with respect to N2. We also separately analysed the mRNA levels of those TGF-β genes at L1, L2 and L4 stages (Figure 1). We chose those stages because of our interest in the TGF-β Dauer and the Sma/Mab pathways, which are supposed to be key in L1-L2 and L4, respectively. The highest mRNA induction was presented by *sma-6*, at any stage. *daf-1* and *daf-4* showed moderate although clearly statistically significant increments at all stages. In relation to *dbl-1*, L1 and L4 but not L2, showed a significant but moderate rise. Surprisingly, in mixed stage we observed the highest *dbl-1* mRNA induction (threefold). Finally, *daf-7* showed a variable stage-dependent increase ranging from no significant change at L2 to fivefold increment at mixed stage. In the cases where *daf-7* is upregulated, the data showed high standard deviations, indicating large variability between the four independent experimental replicas.

### Luciferase reporter assays suggest that miR-58 family members directly regulate TGF-β genes

qPCR experiments can not discriminate between direct and indirect genetic interactions. That is why we ligated the 3′UTR of each candidate gene to a plasmid downstream a luciferase coding sequence, and later transfected them into mammalian cell cultures together with synthetic miR-58, -80, -81 or -1834. All these miRNAs belong to the *mir-58* family, having small differences among themselves (miR-81 and -82 differ only in one nucleotide, and therefore we only used miR-81). *mir-1834* is just known for its presence in the *C. elegans* genome, but there are no mutants available or any other functional feature known about it.

**Figure 2.**
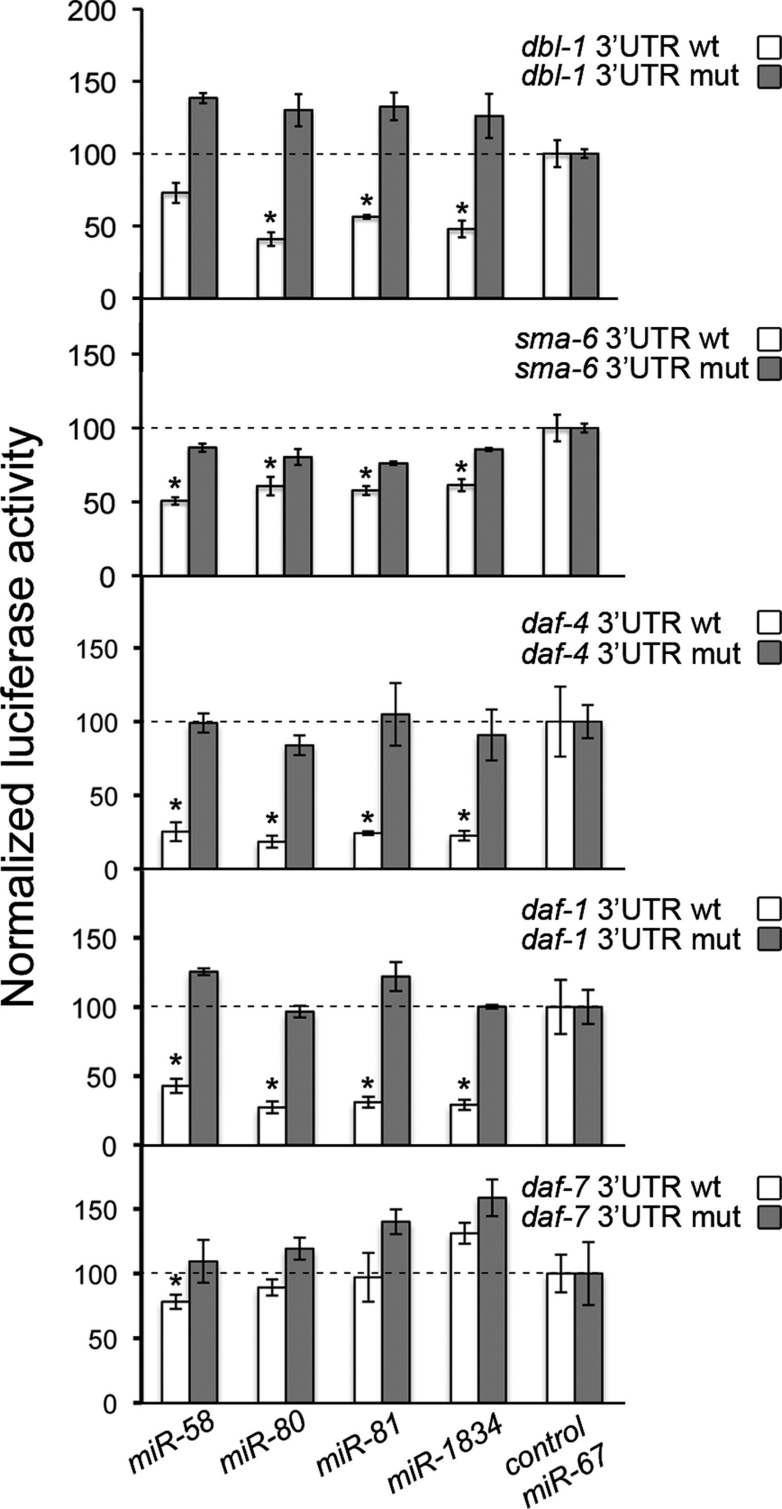
Luciferase reporter assays show that miR-58 family members effectively repress gene expression through the 3′UTRs of TGF-β genes. Human HeLa cells were transiently transfected with psiCHECK-2 vector containing either wild-type (white) or mutated (grey) 3′UTRs from TGF-β genes *dbl-1*, *sma-6*, *daf-4*, *daf-1* and *daf-7*, along with miR-58 family mimics of miR-58, miR-80, miR-81 and miR-1834, or the unrelated miR-67 as negative control. The luciferase activity for each mimic was normalized to the value obtained with miR-67 using the same 3′UTR (dashed line). Data shown are representative of, at least, two independent experiments. Error bars indicate standard deviations. *P < 0.005, comparing the luciferase activity corresponding to each inhibitory miRNA and miR-67.

With the exception of *daf-7* 3′UTR, we confirmed that all the members of miR-58 family (including the uncharacterized miR-1834), but not our negative control miR-67, strongly reduced the expression of the luciferase when placed in front of *dbl-1*, *sma-6*, *daf-4* and *daf-1* 3′UTRs (white bars in Figure 2). This translational repression was dependent on the predicted miRNA binding sites in 3′UTRs, because when these sites were mutated at only three nucleotides the luciferase repression was abolished without exception (grey bars in Figure 2; Supplementary Table S3). Apparently, the 3′UTRs of *daf-1* and *daf-4* are the most efficiently mediators of genetic repression by the miR-58 family, as their means were the lowest for any tested miRNA. The 3′UTR of *sma-6* could also inhibit luciferase expression with any of the miR-58 family members tested. In contrast, *dbl-1* 3′UTR activity seemed more restricted. We did not observe luciferase repression in the case of miR-58. Finally, the 3′UTR of *daf-7* seemed unable to inhibit luciferase gene expression through any of the miRNAs (Figure 2), with the possible exception of miR-58, because of a small but statistically significant difference with respect to control miR-67.

### *In vivo* assays of 3′UTR inhibitory activity confirm that genes of the TGF-β Sma/Mab and Dauer pathways are regulated by miR-58 family

With the possible exception of *daf-7*, all the other assayed TGF-β genes (*dbl-1*, *sma-6*, *daf-4* and *daf-1*) have shown, according to qPCR and luciferase experiments, some degree of downregulation by *mir-58* or other members of its family. In order to confirm a direct regulation of those four genes by the miR-58 family, we performed *in vivo* assays. Thus, transgenic animals carrying an mCherry reporter ligated to every correspondent promoter and 3′UTR were tested in N2 versus a *mir58f(-)* background.

**Figure 3.**
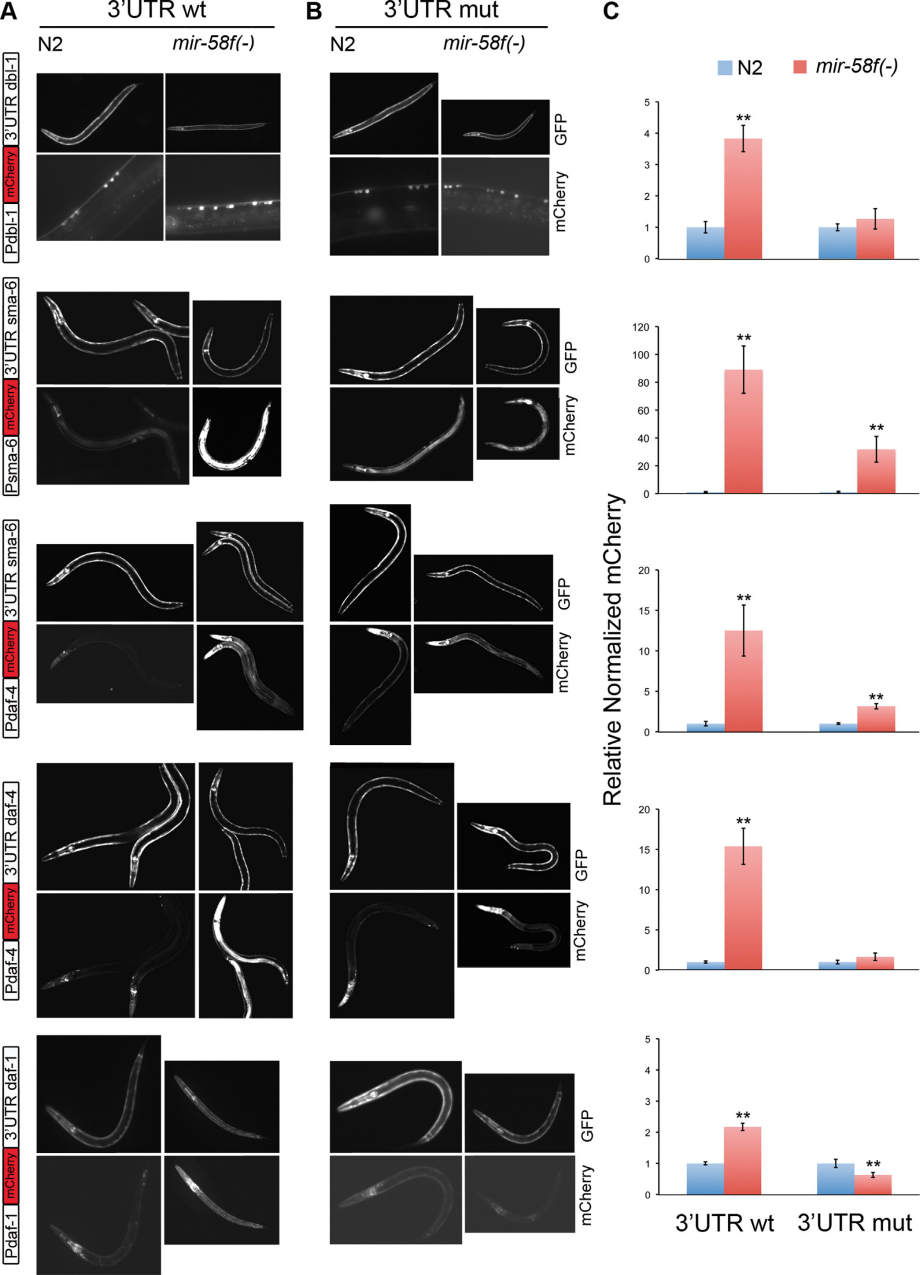
miR-58 family reduces the expression of *dbl-1*, *sma-6*, *daf-4* and *daf-1* through their 3′UTR binding sites *in vivo*. Transgenic worms expressing sensor mCherry constructs (see diagrams to the left) with either the wild-type (**A**) or mutated version of the 3′UTR of each target gene (**B**), namely *dbl-1*, *sma-6*, *daf-4* and *daf-1*, were generated in N2. Two to three independent transgenic lines were crossed into MT15563 (*mir-58f(-)*) to compare mCherry expression between wild-type and *mir-58f(-)* backgrounds. Mean mCherry signal was referred to the mean expression of a coinjected *P_sca-1_::gfp*, to account for varying copy numbers of extrachromosomal arrays. (**C**) Quantification of the normalized mCherry expression using approximately 20 animals per construct and genetic background. Error bars indicate 95% confidence intervals. **P < 0.001, compares normalized mCherry averages between wild-type (blue) and *mir-58f(-)* (red) backgrounds for the same constructs.

As explained in Materials and Methods, to account for spurious oscillations in mCherry intensity, due for instance to differences in extrachromosomal array copies, each transgenic isolate generated in N2 was outcrossed with *mir-58f(-)* worms, to transfer each extrachromosomal array between both backgrounds. Additionally, we coinjected mCherry constructs together with a *P_sca-1_::gfp* DNA in all our assays, so that we could use GFP levels as a control of normalization of the mCherry expression (Figure 3).

**Figure 4.**
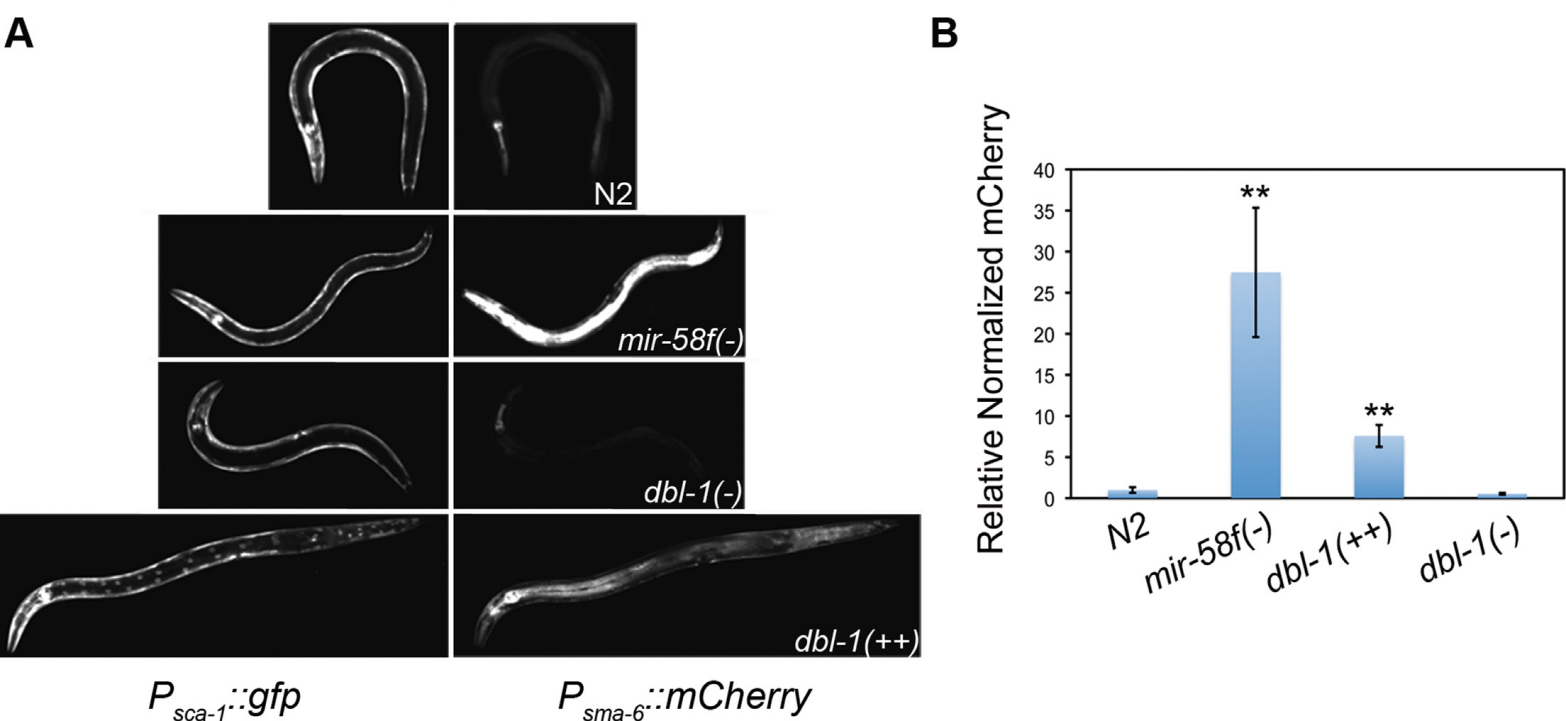
TGF-β Sma/Mab pathway activity is upregulated in the *mir-58* family mutant. A *P_sma-6_::mCherry::3*′*UTR_unc-54_* construct, gene positively regulated by TGF-β Sma/Mab pathway (44,46), was microinjected together with *P_sca-1_::gfp::3*′*UTR_unc-54_* in N2. Two independent transgenic lines were crossed into MT15563 *mir-58f(-)*, NU3 *dbl-1(nk3)* or BW1940 *dbl-1(++)* strains. (**A**) L4 worms showing representative GFP (left panels) and mCherry (right) expression levels in each genetic background. (**B**) Quantification of mean mCherry expression normalized to GFP levels for a minimum of 24 animals per strain. Error bars indicate 95% confidence intervals. **P < 0.001 significance comparing each mutant to N2.

All the four wild-type 3′UTR tested, *dbl-1*, *sma-6*, *daf-4* and *daf-1*, rendered more mCherry activity in a *mir-58f(-)* background than in N2, suggesting that miR-58-family members effectively bind to TGF-β 3′UTRs (Figure 3A and C). However, whereas the repression observed in N2 with the 3′UTRs of *dbl-1* and *daf-1* was moderate (2–4 fold), such repression was much more pronounced with *sma-6* and *daf-4* 3′UTRs (12–15 fold). In relation to *sma-6* we initially observed a massive 90-fold difference (second row from the top of Figure 3). However, it is known that the *sma-6* promoter is positively regulated by the TGF-β Sma/Mab pathway (44,46) and, as we will show below, TGF-β Sma/Mab is in fact more active in *mir-58f(-)* than in N2 worms (Figure 4). Accordingly, to assess the inhibitory activity of the *sma-6* 3′UTR independently from its own promoter, we repeated the same experiment but with the *daf-4* promoter instead. Then, in fact, the difference in mCherry intensity between the N2 and *mir-58f(-)* was not as striking as before (now around 12-fold, third row from the top, Figure 3).

In all cases, when 3′UTRs were mutated in their predicted miR-58f binding sites, the mCherry fluorescence difference between N2 and *mir-58f(-)* disappeared or was largely abolished (Figure 3B and C).

### TGF-β signalling is upregulated in miR-58-family defective worms

Since mRNAs of TGF-β ligands (*dbl-1* and *daf-7*) and all TGF-β receptors (*sma-6*, *daf-4* and *daf-1*) are more abundant in *mir-58f(-)* mutants, we asked whether the two described TGF-β pathways, Sma/Mab and Dauer, are upregulated in such worms.

As already mentioned, the *sma-6* promoter is known to be positively regulated by TGF-β Sma/Mab pathway (44,46). Thus, a *sma-6* transcriptional *P_sma-6_::mCherry* reporter was used to compare the TGF-β Sma/Mab activity between the wild-type and *mir-58f(-)* genetic backgrounds. We also compared with *dbl-1(nk3)* and *dbl-1(++)* backgrounds, as negative and positive controls, respectively. As shown in Figure 4, *mir-58f(-)* mutants revealed much higher *P_sma-6_* activity than N2, and even more than *dbl-1(++)* worms.

**Figure 5.**
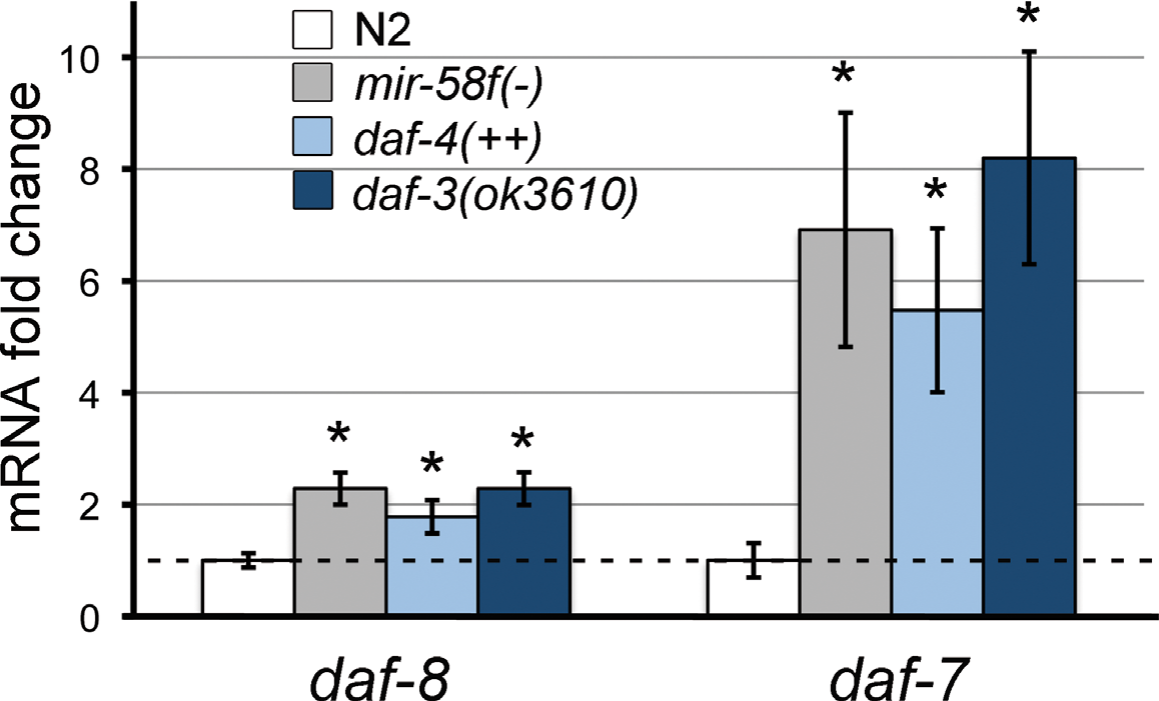
TGF-β Dauer pathway is activated in the *mir-58* family mutant. We used *daf-8* and *daf-7* expression as a sensor of the activity of TGF-β Dauer (45). *daf-8* and *daf-7* mRNA levels were measured by qPCR with specific TaqMan probes in synchronized mixed stage worms of shown mutants compared to wild-type N2 (white bars). Error bars indicate standard deviations, and *P < 0.001 significant differences between each strain and N2. Each value represents the average of four independent experiments.

For the assessment of the activity of the TGF-β Dauer pathway we used *daf-8* and *daf-7* mRNA levels. Both are not only components of the pathway but may also be taken as TGF-β Dauer sensors, as they are transcriptionally repressed by downstream Smad transcription factor DAF-3, which is itself antagonized by the TGF-β Dauer pathway (15). The more active this pathway is, the higher is the activation of both *daf-8* and *daf-7*, which leads to an inhibition of the dauer phenotype (45). Do *mir-58f(-)* worms, unable to enter dauer (5), have higher *daf-8* and *daf-7* mRNA content than N2? We measured *daf-8* and *daf-7* transcripts in mixed-stage by qPCR. As positive control of the pathway activity, we included *daf-3(ok3610)*, a DAF-3 defective worm known to overexpress *daf-8* and *daf-7* mRNAs (45). We also included *daf-4(++)* (47) to find out whether the sole overexpression of the DAF-4 receptor was enough to increase the signalling, similarly as *mir-58f(-)* may do. Figure 5 shows that both, *daf-8* and *daf-7* mRNA levels (especially the second) were significantly elevated in *mir-58f(-)*, and in *daf-4(++)* and *daf-3(ok3610)*, compared to N2. These results are consistent not only with a higher expression of *daf-4* and *daf-1* in *mir-58f(-)* worms, but also with its dauer-defective phenotype.

We conclude that both TGF-β pathways, Sma/Mab and Dauer, are upregulated in the absence of miR-58 family members.

### miR-58 family influences body size both dependent and independently of TGF-β Sma/Mab signalling pathway

As described by Alvarez-Saavedra and Horvitz (5), the *mir-58* family mutant presents a highly reduced body size, similar in length to TGF-β Sma/Mab depleted animals. To tackle this issue we first aimed to study how each of the *mir-58* family members contributes to adult body size. Therefore, we measured the length of worms carrying each of the three null mutations present in *mir-58f(-)* separately, and also the corresponding three double mutants. We observed that neither *mir-80(nDf53)*, nor *mir-81&mir-82(nDf54)*, or a combination of both, showed any significant reduction in length with respect to N2 (Table 1). However, *mir-58(n4640)* showed such a reduction (*P* < 0.001), which was even more pronounced in the company of *mir-80(nDf53)* (*P* < 0.001) but not of *mir-81&mir-82(nDf54)* (*P* = 0.2). When the three deletions were present the length reduction was even larger (*P* < 0.001 with respect to *mir-58(n4640)* alone or together with any of the other two mutations). We conclude that miR-58 is the miRNA that contributes the most on body size, followed by miR-80 and the tandem miR-81/-82 (in that order).

**Table 1.** *mir-58* family affects body size differently and independently of TGF-β Sma/Mab

Genotype	% length ± 95% CI	N	*P*
N2	100 ± 0.7	139	
*mir-81&mir-82(nDf54)*	100.3 ± 1.4^a^	89	0.56^a^
*mir-80(nDf53)*	98.6 ± 1.0^a^	79	0.19^a^
*mir-80(nDf53);mir-81&mir-82(nDf54)*	99.8 ± 1.2^a^	48	0.12^a^
*mir-58(n4640)*	87.8 ± 0.9^a^	130	<0.001^a^
*mir-58(n4640);mir-81&mir-82(nDf54)*	82.2 ± 1.6^a^	52	0.20^c^
*mir-58(n4640);mir-80(nDf53)*	78.5 ± 1.0^a^	49	<0.001^c^
*mir-58(n4640);mir-80(nDf53);mir-81&mir-82(nDf54)*	60.4 ± 1.2^a^	49	<0.001^c,d,e^
*dbl-1(nk3)*	63.8 ± 1.1^a^	40	0.33^f^
*mir-58(n4640);dbl-1(nk3)*	49.5 ± 1.1^a^	39	<0.001^c,g^
*ctIs40(dbl-1++)*	114.2 ± 2.3^a^	36	
*mir-58(n4640);ctIs40(dbl-1++)*	105.8 ± 2.8^a^	20	0.002^h^
			
*mir-58(n4640);mir-80(nDf53);mir-81&mir-82(nDf54);vector(RNAi)*	100 ± 1.0	90	
*mir-58(n4640);mir-80(nDf53);mir-81&mir-82(nDf54);sma-6(RNAi)*	76.9 ± 1.2^b^	73	<0.001^i^
*mir-58(n4640);mir-80(nDf53);mir-81&mir-82(nDf54);daf-4(RNAi)*	96.0 ± 1.3^b^	30	<0.001^i^
			
N2 (bis)	100 ± 1.5	39	
*sma-6(wk7)*	67 ± 1.9^j^	58	<0.001^j^
*sma-6(wk7);sma-6::3*'*UTR sma-6 WT*	105.0 ± 2.1^j^	50	<0.001^j^
*sma-6(wk7);sma-6::3*′*UTR sma-6 MUT*	112.1 ± 1.4^j^	50	<0.001^j,k^
*mir-58f(-)*	100 ± 1.9	40	
*mir-58f(-);sma-6::3*′*UTR sma-6 WT*	111.0 ± 2.1^l^	60	<0.001^l^
*mir-58f(-);sma-6::3*′*UTR sma-6 MUT*	108.8 ± 1.4^l^	40	<0.001^l^,
			0.486^m^

N number of measured worms.

^a^Compared to N2.

^b^Referred to the length of *mir-58(n4640);mir-80(nDf53);mir-81&mir-82(nDf54)* treated with RNAi empty vector.

^c^Compared to *mir-58(n4640)*.

^d^Compared to *mir-58(n4640);mir-81&mir-82(nDf54)*.

^e^Compared to *mir-58(n4640);mir-80(nDf53)*.

^f^Compared to *mir-58(n4640);mir-80(nDf53)*;*mir-81&mir-82(nDf54)*.

^g^Compared to *dbl-1(nk3)*.

^h^Compared to *ctIs40(dbl-1++)*.

^i^Compared to empty vector (RNAi).

^j^Compared to N2 (bis).

^k^Compared to *sma-6(wk7);sma-6::3*′*UTR sma-6 WT*.

^l^Compared to *mir-58f(-)*.

^m^Compared to *mir-58f(-);sma-6::3*′*UTR sma-6 WT*.

We then asked whether double mutants for the TGF-β Sma/Mab pathway and the miR-58 family, both of a similar length on their own, would reach a similar size or become even smaller. Our results showed that the double mutant *mir-58(n4640);dbl-1(nk3)* was significantly shorter than worms with either mutation alone (*P* < 0.001; Table 1). *mir-58(n4640);ctIs40(dbl-1++)* animals presented an intermediate size between the shorter *mir-58(n4640)* and the longer DBL-1 overexpressing worm *ctIs40(dbl-1++)*. As an alternative approach, we inhibited two Sma/Mab genes by RNAi, *sma-6* and *daf-4*, in a *mir-58f(-)* background. The depletion of any of those two genes was able to further diminish the length of *mir-58f(-)* (Table 1). We conclude that the effect of the miR-58 family on body size is at least partly independent of TGF-β Sma/Mab pathway.

**Figure 6.**
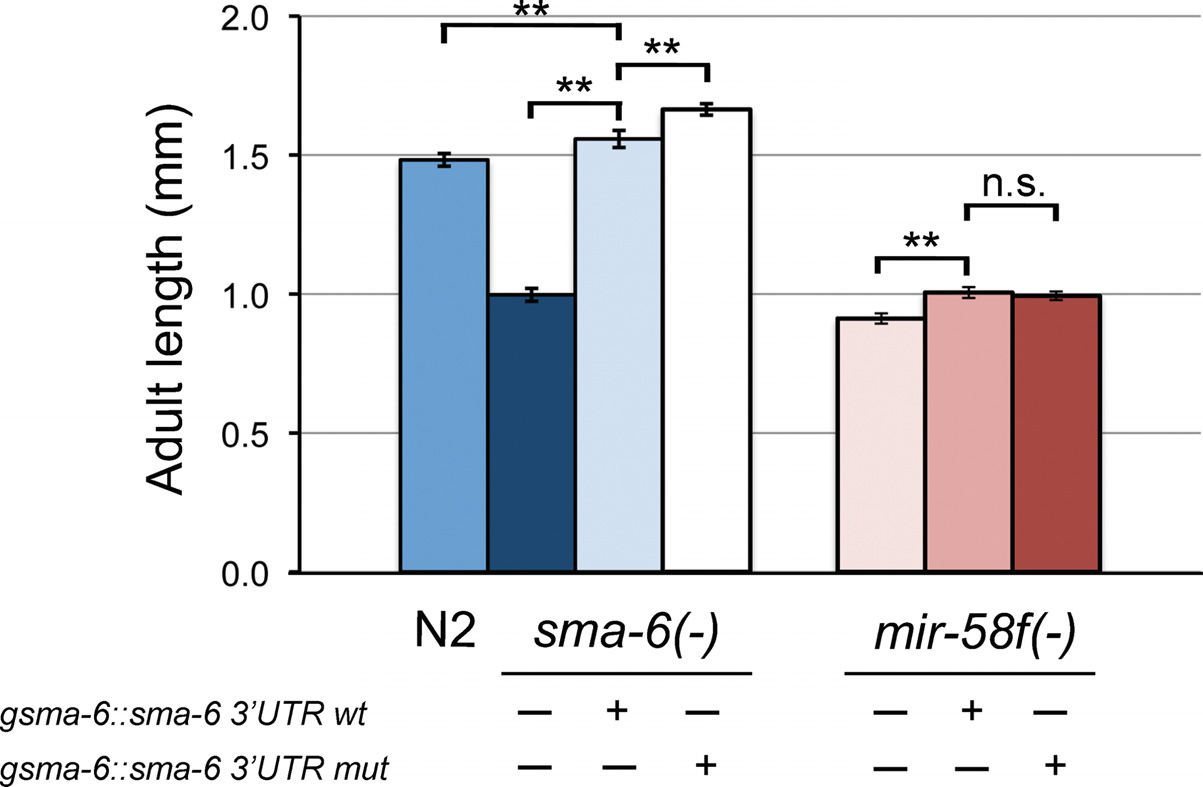
Inactivation of miR-58f binding sites on *sma-6* 3′UTR makes *C. elegans* longer. Adult worm length was estimated in animals that overexpressed genomic *sma-6* with either wild-type or mutated 3′UTR in *sma-6(wk7)* defective background (A) or *mir-58f(-)* worms (B). Data represent the average of two independent experiments. Error bars indicate 95% confidence intervals and significant statistical differences between strain average length are indicated as **(*P <* 0.001).

Next, we tested and quantified the presumptive inhibitory effect of *mir-58f* on growth through the TGF-β Sma/Mab pathway, without the opposite (i.e. positive) effects on growth via other genes or signalling pathways. For that purpose, a *sma-6* construct, that includes its 3′UTR either wild-type or mutated in its putative *mir-58f* binding sites, was microinjected in *sma-6(wk7)* defective worms. Overexpression of *sma-6* with the mutated 3′UTR makes worms 12% longer than N2 (*P* < 0.001), whereas the same construct but with the wild-type *sma-6* 3′UTR results in smaller worms (only 5% longer than N2; *P* < 0.001) (Figure 6 and Table 1). Importantly, the 7% difference in favour of the worms overexpressing *sma-6* with mutated 3′UTR, compared to those with a wild-type 3′UTR, is statistically significant (*P* < 0.001). As negative control, both *sma-6* constructs were microinjected in *mir-58f(-)* mutants. We expected to detect no difference in length between worms carrying the wild-type 3′UTR extrachromosomal array and those expressing the mutant 3′UTR construct. Indeed no significant difference was found between both 3′UTRs (Figure 6 and Table 1). These results confirm that, independently of its promoting effects on growth through other pathways, *mir-58f* also inhibits growth through TGF-β Sma/Mab.

### miR-58 family mutants do not enter dauer due to upregulation of TGF-β Dauer pathway

**Figure 7.**
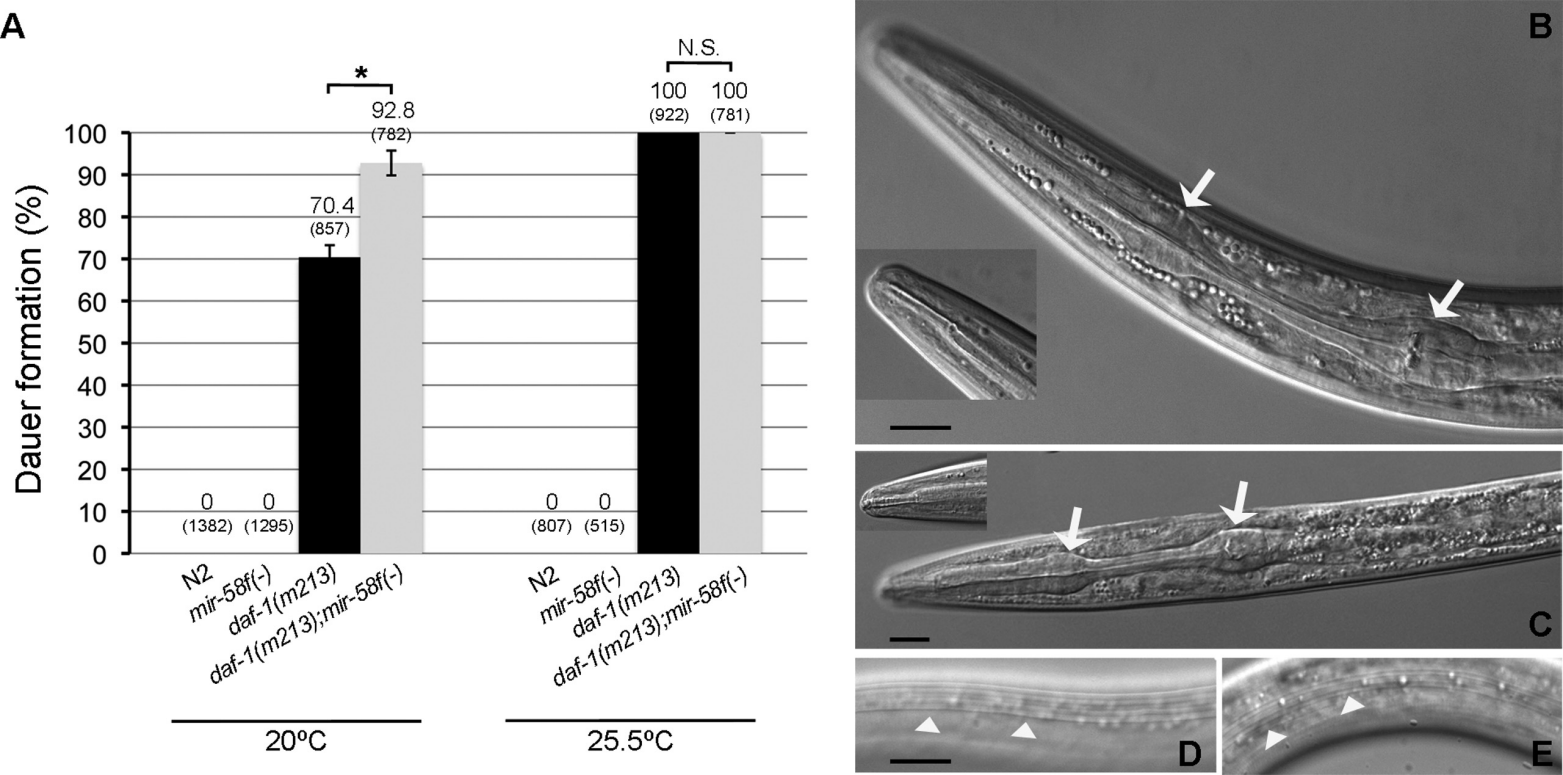
*daf-1* is epistatic to *mir-58* family for the dauer-deficient phenotype. (**A**) Dauer assays were performed at 20 and 25.5ºC on 5 independent plates per strain. Number above the bars shows the percentage of dauers and the numbers in parentheses indicate the total number of animals scored. Error bars indicate 95% confidence intervals. Statistical evaluation is represented as N.S.(not significant) or *(*P =* 0.001). Confocal images of the pharynges of dauers in *daf-1(m213)* (**B**), *mir-58f(-);daf-1(m213)* (**C**). Normarski micrographs of the lateral alae in *daf-1(m213)* dauer (**D**) and *mir-58f(-);daf-1(m213)* dauer (**E**). Scale represents 10 μm. Arrows indicate the radial constriction in the pharynx, which is similar in both *daf-1(m213) and mir-58f(-);daf-1(m213)* dauers. Arrowheads show dauer alae present in both genotypes.

Another interesting phenotype of *mir-58f(-)* is its inability to enter the dauer stage (5). Could that be related to our finding that the TGF-β Dauer pathway, which is dauer inhibitory, is upregulated in *mir-58f(-)*? We blocked the TGF-β Dauer pathway in *mir-58f(-)* worms by impairing *daf-1*, and ask whether dauer formation could be rescued. Figure 7A shows that *mir-58f(-);daf-1(m213)* mutants produced an even higher percentage of dauers than *daf-1(m213)* at 20ºC, whereas both genotypes gave a 100% of dauers at 25.5ºC, fully overturning *mir-58f(-)*'s dauer deficiency. We did not observe additional phenotypes, such as embryo or larval lethality. To verify the presence of true dauers in *mir-58f(-);daf-1(m213)* plates, we first looked at their sodium dodecyl sulphate (SDS) resistance, which confirmed them as dauers. Secondly, we looked for alae structures in animals looking like dauers. As seen in Figure 7E, *mir-58f(-);daf-1(m213)* exhibited alae very similar to those of *daf-1(m213)* dauers (Figure 7D), whereas, as described, they were missing from *mir-58f(-)* (5). Thirdly, we compared the pharynx morphology of *mir-58f(-);daf-1(m213)* and *daf-1(m213)* and found that both had constricted pharynges and sealed mouths, as expected for dauers (Figure 7C and B, respectively). In summary, our results indicate that *mir-58f(-)* is unable to form dauers because of an hyperactivation of the TGF-β Dauer pathway.

**Figure 8.**
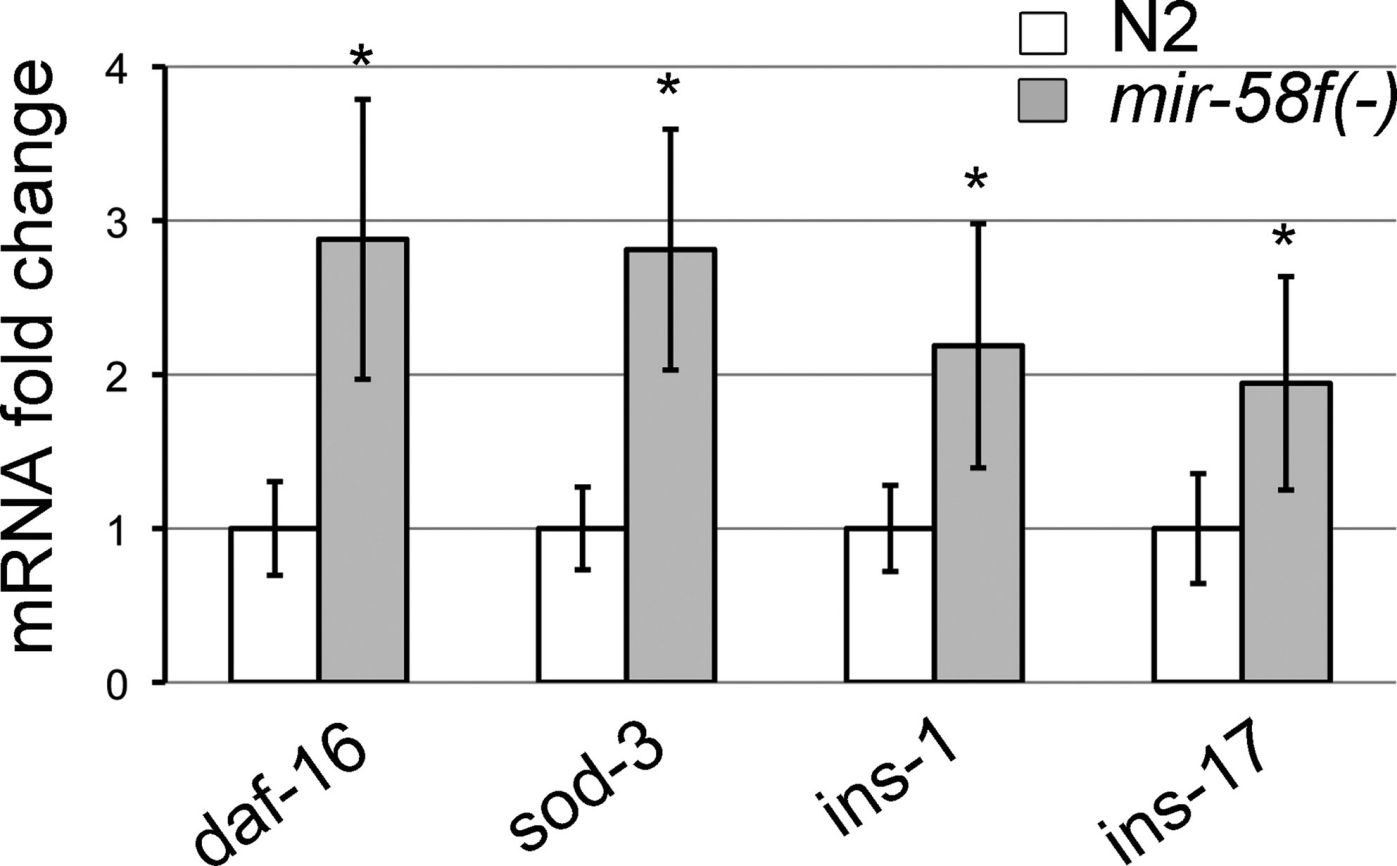
*sod-3, daf-16, ins-1* and *ins-17* mRNAs are elevated in *mir-58* family mutants. qPCR of *sod-3*, *daf-16*, *ins-1* and *ins-17* in L1 stage population of *mir-58f(-)* versus N2. It represents the average of four independent experiments. Error bars indicate standard deviations. Significant statistical differences between N2 and *mir-58f(-)* are indicated as *(*P <* 0.001).

Could other molecular routes be involved as well? Although we have just showed that an upregulation of TGF-β Dauer is enough to explain the dauer-deficient phenotype of *mir-58f(-)* (Figure 7), we also checked the insulin/IGF-1 signalling (IIS) pathway. First, we gauged the activity of IIS in *mir-58f(-)* animals using *sod-3* as reporter. Because IIS is dauer inhibitory and *sod-3* is repressed by IIS, if IIS was responsible of the dauer deficiency of *mir-58f(-)*, we would expect IIS to be upregulated and *sod-3* mRNA to be downregulated in *mir-58f(-)*. In contrast, we found a nearly threefold increment in *sod-3* transcript levels in *mir-58f(-)* L1 worms compared to N2 (Figure 8). Additionally, we found *daf-16, ins-1* and *ins-17* also upregulated in *mir-58f(-)* (Figure 8). Because the dauer stage depends on the presence of DAF-16 (48), and INS-1 and INS-17 are insulin antagonists (49,50), our results clearly show a downregulation of IIS activity in *mir-58f(-)*, which again is inconsistent with a responsibility of IIS in the dauer deficiency of *mir-58f(-)*. Furthermore, we produced a *mir-58f(-);daf-2(e1370)* mutant (DAF-2 is the receptor of IIS), and observed that it had a proportion of arrested larvae which resembled dauers (Supplementary Table S6). However, after close inspection, we observed that they were able to pump, that they were unable to survive 1% SDS treatments, that they lacked the characteristic cuticle *alae* of true dauers, and that their mouths were open and their pharynges unconstricted (Supplementary Figure S1). We conclude that *mir-58f(-);daf-2(e1370)* worms do not enter dauer and that IIS does not mediate *mir-58f(-)* dauer defective phenotype.

### TGF-β Sma/Mab positively regulates *mir-58* transcription

**Figure 9.**
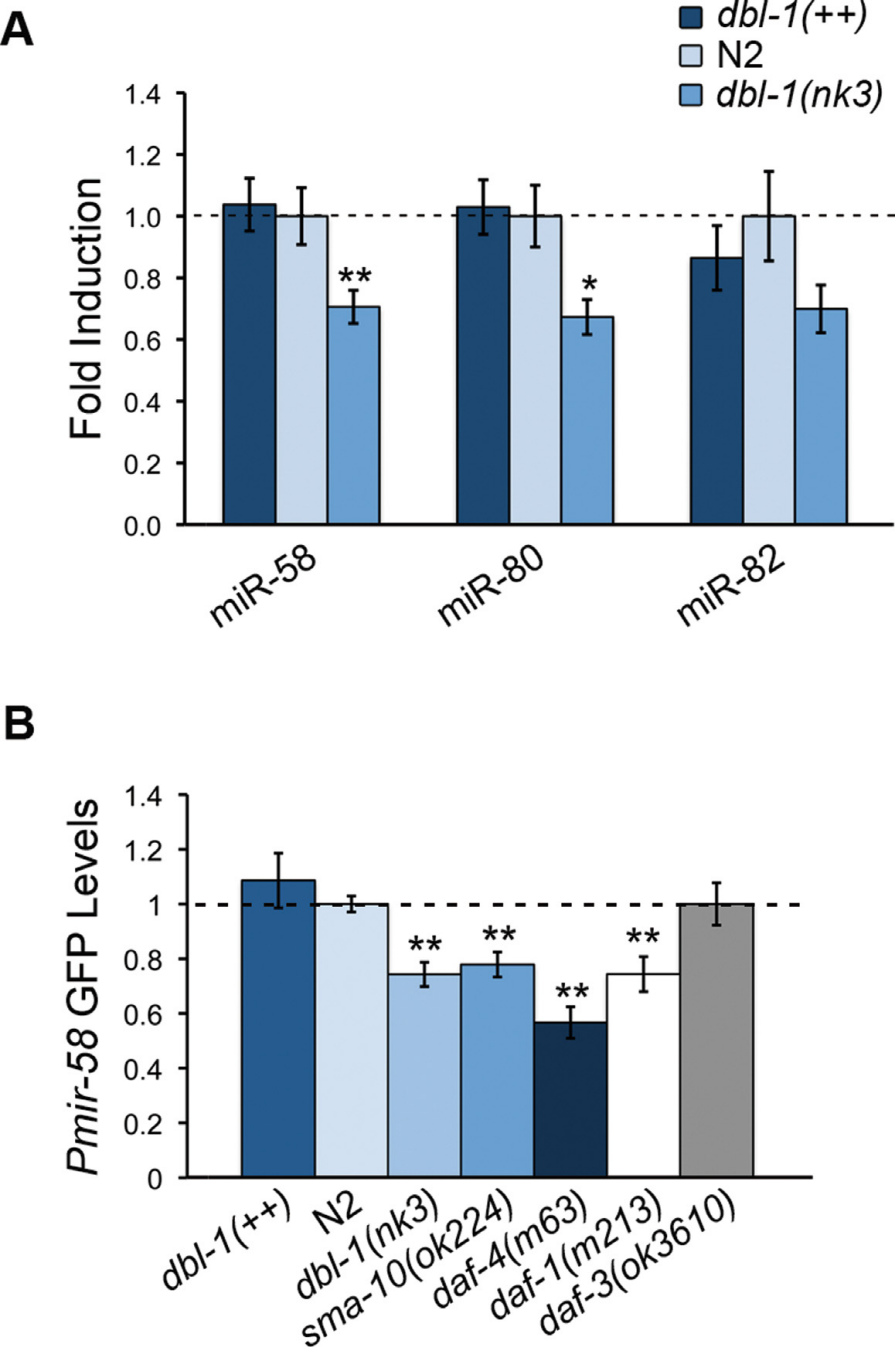
Deficient TGF-β Sma/Mab and Dauer pathways reduce the expression of *mir-58* family. (**A**) qPCR of mature miR-58, miR-80 and miR-82 in *dbl-1(++)*, N2 and *dbl-1(nk3)* L4 worms. It represents the average of two independent experiments. Error bars indicate standard deviations. *P < 0.01 and **P < 0.001 show significant differences between each strain and N2. (**B**) Expression of *P_mir-58_*::*gfp* in *dbl-1(++)*, *dbl-1(nk3)*, *sma-10(ok2224)*, *daf-4(m63)*, *daf-1(m213)* and *daf-3(ok3610)*, all of them normalized to N2 levels (EUB0032 strain; dashed line). Error bars indicate 95% confidence intervals. A range of 27–157 animals was measured per strain. **P < 0.001, compared to N2.

In Drosophila the Dpp pathway (TGF-β) controls *bantam* (*mir-58* homolog) expression (32–34). This led us to ask whether TGF-β Sma/Mab and/or TGF-β Dauer may regulate *mir-58* transcription. Figure 9A shows the levels of miR-58, miR-80 and miR-82 in *dbl-1(++)* and *dbl-1(nk3)* relative to N2, in synchronized L4 worms. Around a 30% decrease of each miRNA in *dbl-1(nk3)* mutants was noticed, although no increment in the *dbl-1(++)* strain was observed. We detected a significant difference between N2 and *dbl-1(nk3)* at miR-58 (*P* = 0.001) and miR-80 (*P* = 0.007), but not at miR-82 (*P* = 0.078). This could be due to the observed larger variation in miR-82 levels in *dbl-1(nk3)* and particularly in N2 animals.

Additionally, we used a *P_mir-58_::gfp* reporter to assess its transcriptional activity in various TGF-β altered backgrounds (Figure 9B). We noticed an approximate 20–40% reduction, compared to N2 (*P* < 0.001), in three TGF-β Sma/Mab deficiency mutants: *dbl-1(nk3)*, *sma-10(ok2224)* and *daf-4(m63)*. These results confirm that the absence of TGF-β Sma/Mab downregulates the *mir-58* family.

Using the same two previous approaches, we assessed the transcriptional levels of *mir-58* in the context of TGF-β Dauer pathway. We found that the downregulation of TGF-β Dauer receptors DAF-1 and DAF-4 (DAF-4 is shared between both TGF-β pathways) led to a decrease in *P_mir-58_* activity in L4 (Figure 9B). However, no *P_mir-58_* increase occurred when the negative regulator *daf-3(ok3610)* was tested (Figure 9B) (15). More importantly, no significant variation of the levels of miR-58 family members were detected in L1 larvae when the TGF-β Dauer pathway was either downregulated (in *daf-1(m213)* and *daf-4(m63)* backgrounds) or upregulated (with *daf-3(ok3610)* and *P_daf-8_::daf-8::gfp*; Supplementary Figure S2). Therefore, our results do not really support that *mir-58* expression depends on TGF-β Dauer pathway activity, although we do not exclude it either.

## DISCUSSION

**Figure 10.**
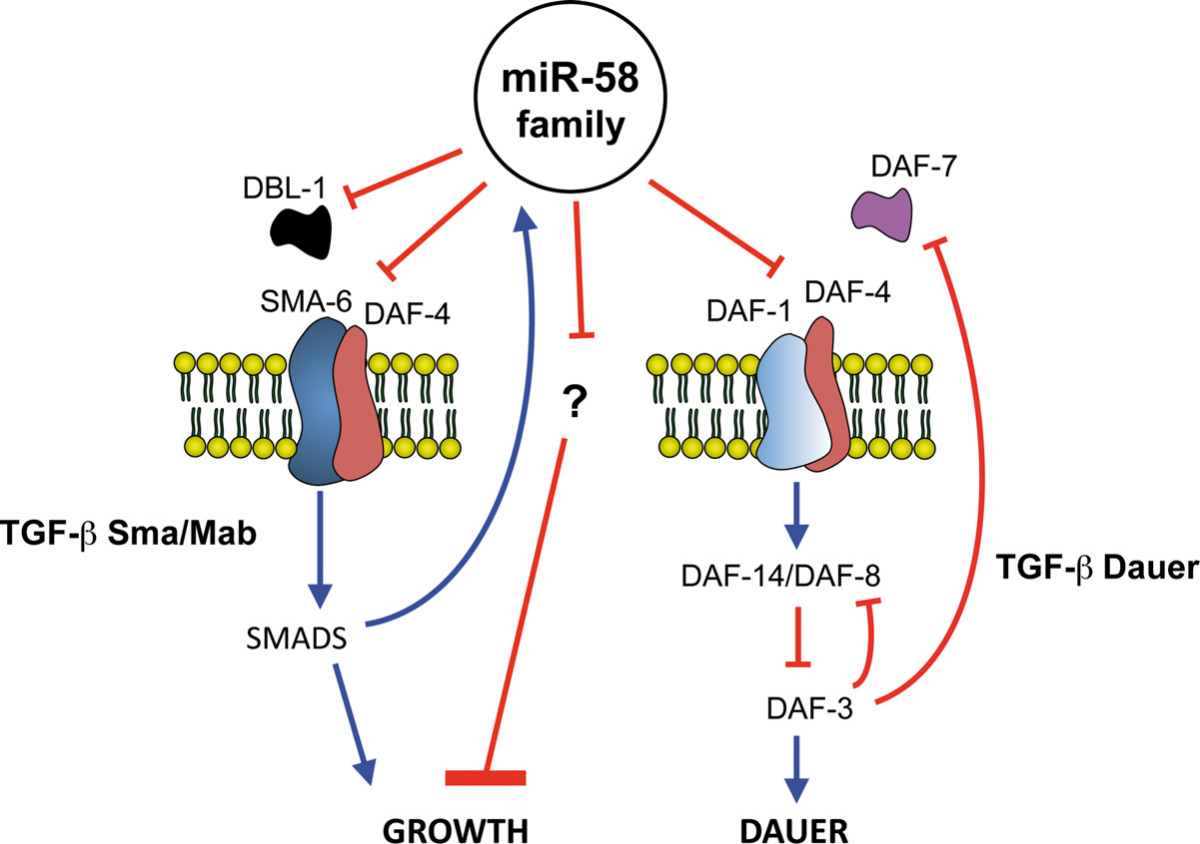
Model for regulation of growth and dauer by miR-58 family. *mir-58* family downregulates both TGF-β pathways, Sma/Mab and Dauer. In the Sma/Mab branch, this inhibition goes through ligand DBL-1 and receptor proteins SMA-6 and DAF-4. Therefore, miR-58 family activity is growth-inhibitory through Sma/Mab. In parallel, it also acts as a larger growth-promoting signal, by repressing unknown growth inhibitors. Additionally, TGF-β Sma/Mab positively regulates the transcription of *mir-58* and *mir-80*, thus creating a negative feedback loop. In the TGF-β Dauer pathway, the miR-58 family targets receptors DAF-1 and DAF-4. This inhibition shall inactive DAF-14/DAF-8 dimers, which will allow the activation of downstream antagonistic DAF-3. Through DAF-3, DAF-8 and DAF-7 will be transcriptionally downregulated and therefore dauer entry will be promoted. In summary, the miR-58 family modulates both TGF-β routes, acting as a new hub that may coordinate growth and dauer decisions.

One way to facilitate the identification of miRNA targets is to study miRNA-family mutants as opposed to single mutants. In this way, we are more likely to observe abnormal phenotypes as well as interactions between those miRNAs and other loci. This is what we have done for the *mir-58* family (or *mir-58f*), by using the strain MT15563 (*mir-58f(-)*), a *C. elegans* strain missing four members of that family: *mir-58*, *-80*, *-81* and *-82* (5). Our results are summarized in the model of Figure 10.

### Control of TGF-β genes by the *mir-58* family

Our gene reporter assays strongly suggest that *mir-58f* directly downregulates four genes (*sma-6*, *daf-4*, *daf-1* and *dbl-1*) from the TGF-β signalling pathways, Sma/Mab and Dauer, in *C. elegans* (Figures 2 and 3). Two recent papers (51,52), using high-throughput genomic methods, support our conclusion that miR-58f repress the mRNA levels of those genes through direct interactions with their corresponding 3′UTRs. In the more recent approach (51), they cross-link, ligate and sequence miRNA-mRNA hybrids to ‘unambiguously identify miRNA:target site interactions’. Searching their data set, we found that miR-58, -80, -81 and -82, they all bind to a region in chromosome III that corresponds to the *daf-4* 3′UTR, and that perfectly match two of the three sites that we identified as target regions of miR-58 family (Supplementary Table S5). Using a second method (51,52), they sequenced thousands of 3′UTR fragments associated to Argonaute (ALG-1), and for this reason likely targets of miRNA regulation. In this way, they found three different 3′UTR fragments of *sma-6* (57 bp the smallest) that contain our predicted miR-58f-binding sites. They also found two sequences of 47 bp and 162 bp, from the *dbl-1* and *daf-1* 3′UTRs, respectively, again containing our predicted miR-58f-binding sites (Supplementary Table S5). In summary, recent high-throughput results provide supporting evidence for a direct physical association between miR-58f and the 3′UTRs of *sma-6*, *daf-4, daf-1* and *dbl-1*. Of course, our results go beyond the mere description of those physical interactions and present some of their unfolding consequences in relation to gene expression, cell signalling and phenotypes.

It is interesting that for both, TGF-β Sma/Mab and Dauer, we find that miR-58f is acting at the beginning of their signalling cascades, at the ligand/receptor level (Figures 2 and 3). Perhaps that has been a feature evolutionarily selected on the basis of higher biochemical efficiency. However, our luciferase assays do not really support TGF-β Dauer ligand mRNA, *daf-7*, as direct target of miR-58f (Figure 2). We think that the overexpression of TGF-β Dauer receptor DAF-1/DAF-4, in *mir-58f(-)* animals, might trigger a positive feedback loop that would eventually increase the levels of DAF-7. In this way, an overexpression of TGF-β Dauer receptor would activate downstream Smads DAF-14/DAF-8, which in turn would inhibit another Smad, DAF-3, that is a transcriptional repressor of *daf-7* and *daf-8* (45). In consequence, perhaps an even small increment of *daf-4* and *daf-1* transcripts could lead to a circular self-reinforcement of the TGF-β Dauer pathway, giving rise in the process to increasing levels of *daf-7* mRNA. Two of our results especially support this hypothesis. First, that the TGF-β Dauer pathway appears more active in *mir-58f(-)* than in N2, and with high levels of *daf-7* mRNAs (Figures 1 and 5). Second, that the experimental overexpression of *daf-4* alone suffices to trigger an activation of the TGF-β Dauer pathway, again involving higher expression of the endogenous *daf-7* gene, similarly to what happens in *mir-58f(-)* (Figure 5).

*sma-6* is the most overexpressed mRNA at any developmental stage (Figure 1). We think that this is due to a combination of direct and indirect effects on *sma-6* by *mir-58f*. Thus, it is known that the *sma-6* promoter is positively regulated by its own TGF-β Sma/Mab pathway (44,46). In agreement with that, when we substituted *P_sma-6_* by *P_daf-4_*, in *mCherry::3*′*UTR_sma-6WT_* construct in our *in vivo* reporter assays, there was an 7-fold reduction in mCherry overexpression in *mir-58f(-)* worms. In spite of that reduction, the intensity of *P_daf-4_::mCherry::3*′*UTR_sma-6WT_* was still 15 times higher in *mir-58f(-)* than in N2 (Figure 3). Therefore, both the 5′-flanking region of *sma-6* and its 3′UTR, must be responsible for upregulating *sma-6* mRNA in the quadruple *mir-58f(-)* mutant (Figure 1). The overexpression of the other ligand in *mir-58f(-)*, *daf-7*, is irregular but generally high nonetheless (Figure 1). As previously discussed, that is most likely due to transcriptional activation (45).

### Control of adult growth by *mir-58* family

The most striking and recognizable feature of *mir-58f(-)* worms is their small body size (5). However, overexpression of DBL-1 results in longer, not shorter, animals (8,9). Therefore, it appears that the upregulation of the TGF-β Sma/Mab pathway in *mir-58f(-)* worms can not explain their small body size. In agreement with that, our epistasis experiments show that the effects of *mir-58f* and TGF-β Sma/Mab on body size are at least partly independent from each other (Table 1), suggesting that other pathways affecting growth are regulated by *mir-58f*.

The fact that the worms with *sma-6* 3′UTRs altered in key miR-58f recognition sites were 7% longer than those with wild-type 3′UTRs leaves little doubt that miR-58f inhibits worm length through *sma-6* (and presumably *daf-4* and *dbl-1* as well; Figure 6 and Table 1). Therefore, independently of its stimulating effect on body size through unknown growth-inhibitory genes, *mir-58f* also has an inhibitory influence on growth derived from its interaction with TGF-β Sma/Mab. Since *mir-58f(-)* worms are much smaller than N2, of course these two antagonistic actions are not equivalent. But perhaps subtle spatiotemporal differences in gene expression of either the target genes, and/or the *mir-58f* members, can modulate those two opposing effects, at times giving way to a predominantly inhibitory role to miR-58f.

In relation to the overall effect on growth of each miR-58 family member, we have shown that their contribution to body length is highest for miR-58, intermediate for miR-80 and lowest for miR-81 & -82 (Table 1). One possibility is that this is due to their relative abundances, which generally fits well with their expression levels (20,21). However, these miRNAs also differ on their expression patterns, and then we should expect some degree of specialization in relation to body size and other functions (22,23). As a first attempt to understand the relative importance of the various organs in relation to body size we performed rescue experiments using promoters specific of two of the tissues where miR-58 is present, gut and hypodermis, in *mir-58f(-)* worms. We observed an approximate 25% length recovery for each promoter (Supplementary Figure S3). Therefore, we suspect that miR-58 acts on body size through a combination of tissues.

We may speculate that an additional tissue involved could be the germline. We know that the germline inhibits body growth because when that tissue is ablated in larvae, adults become larger (53,54). Interestingly, it has been recently shown that *mir-58f* motifs are underrepresented in the germline transcriptome, suggesting a strong regulation by *mir-58f* at this tissue (55).

It is also interesting to compare worms with fruitflies in relation to miR-58f and growth. As previously mentioned, *bantam* is the sole *mir-58* Drosophila ortholog, and the correspondent loss-of-function flies are small because of a reduction in cell number but not cell size (24,28). Then, we checked whether *mir-58(-)* nematodes also have a proliferation defect, but actually these mutants contain the same number of hypodermal nuclei at adulthood as N2 do (Supplementary Figure S4). We looked at the hypodermis because it is largely responsible for adult growth (10). In spite of those differences, Drosophila might also guide us to look for miR-58f target genes in miR-58f's growth-promoting task. It is possible that some of the miRNA targets were shared across far-related species, as it is the case for other miRNAs, like *let-7* and two of its targets, RAS (56) and *lin-41*, present in Drosophila and vertebrates (57). Five targets of *bantam* have been experimentally validated so far, i.e. *mei-P26*, *hid*, *enabled*, *capicua* and *Socs36E* (58). *ncl-1* is *C. elegans*’ counterpart of *mei-P26*, and also a predicted *mir-58f* target by specialized software. Mei-P26 / NCL-1 is a zinc finger protein that is involved in cell proliferation and growth through the regulation of dMyc activity in Drosophila, whereas in *C. elegans* is thought to repress ribosome synthesis (59). That is consistent with the small phenotype of *mir-58f(-)*, because then NCL-1 would be expected to inhibit growth.

### Role of *mir-58* family in dauer formation

Our finding that the TGF-β Dauer pathway, which represses dauer formation, is upregulated in *mir-58f(-)* opens up the question of whether it is that upregulation what makes the *mir-58f(-)* strain dauer defective. Our epistasis analyses support this view, because the quintuple mutant *mir-58f(-);daf-1(m213)* is dauer constitutive, as *daf-1(m213)* is, in consequence placing *daf-1* downstream from *mir-58f*. In fact, the percentage of dauers at 20ºC is even larger in *mir-58f(-);daf-1(m213)* than in *daf-1(m213)*, i.e. 93% versus 70%, respectively (Figure 7). One possibility why this might be so is that not all the four members of *mir-58f* behaved as dauer facilitators. Thus, one of them could be dauer repressor acting through a different signalling pathway, although masked by the action of the facilitators. If that was correct, when the TGF-β Dauer signalling pathway becomes inactive, like in a *daf-1(m213)* background, the only miRNA working independently from TGF-β Dauer pathway would be the dauer-repressor miRNA. As a result, there would be more dauers in *mir-58f(-);daf-1(m213)* than in *daf-1(m213)*, as we observe. In accordance with this hypothesis, a recent paper by Than et al. suggests that *mir-81*, in contrast with the rest of its family, is a dauer repressor acting through the cyclic guanosine monophosphate signalling (cGMP) (60). On the other hand, miR-81 is much less expressed than miR-58 and -80 (21), and then this may restrict its potential to act as a dauer inhibitor in the company of the other members of the family. Also, in support of the above, we know that miR-81, in contrast to miR-58, -80 and -82, is the only one that is not able to rescue the dauer defective phenotype of *mir-58f(-)* when expressed by itself (5).

Although we can explain *mir-58f(-)* dauer defectiveness on the basis of *daf-7*, *daf-1* and *daf-4* overexpression, it would be possible that other *mir-58f* target genes may also be implicated in dauer regulation, just because individual miRNAs, let alone families, are supposed to control multiple genes (61). We investigated whether the insulin/IGF-1 signalling (IIS) pathway might be necessary for the dauer-defective phenotype of *mir-58f(-)*, and we found that it is not. First, it is downregulated in *mir-58f(-)* (Figure 8) and that alone should favour dauer formation (i.e. IIS is dauer inhibitory). Second, the suppression of the insulin pathway in *mir-58f(-)* worms does not elicit the development of true dauers. Instead, *mir-58f(-);daf-2(e1370)* worms at 25ºC generate arrested larvae often resembling dauers but without their key features (i.e. cuticle alae, sealed buccal cavity, constricted pharynx; Supplementary Figure S1, Table S6). Rather, it seems that *mir-58f(-);daf-2(e1370)* show an intermediate phenotype with respect to *mir-58f(-)* and *daf-2(e1370)*. This gives support to the idea of a parallel signalling for miR-58f / TGF-β Dauer on the one hand and IIS on the other, with respect to dauer, which is in agreement with the existing literature as far as the TGF-β Dauer and IIS pathways are concerned (62). Recently Vora et al. observed that *mir-80(-)* worms are healthier and live longer than N2, and that this phenotype is *daf-16* dependent (27). That is consistent with our results because the activities of DAF-16 and IIS antagonize each other (63), and therefore one could expect that *mir-80(-)* presented a somewhat downregulated IIS, which is what we observe in *mir-58f(-)* (Figure 8).

### Autoregulatory feedback loops

We have shown that not only *mir-58f* negatively controls TGF-β Sma/Mab, but also that this pathway stimulates the transcription of *mir-58* and *mir-80*. Therefore, a negative feedback loop is established between both molecular sets. However, the two less abundant members of *mir-58f*, miR-81 and -82 (21), do not significantly change their expression depending on the TGF-β Sma/Mab pathway (Figure 9A). Nor have we observed substantial miRNAs upregulation if the TGF-β Sma/Mab pathway is overexpressed, only a consistent miRNA fall when TGF-β Sma/Mab is disrupted (Figure 9). Perhaps the overexpression of *dbl-1(++)* that we used exceeds what may be physiological in the wild-type, and that is why the miR-58/-80 promoters do not have a natural response for it.

The control over *mir-58* and *mir-80* could be transcriptional or posttranscriptional. In mammals there are examples of both forms of regulation between miRNAs and TGF-β signalling (64). Our system appears transcriptional because of our results with a *P_mir-58_::gfp* reporter (Figure 9B). Moreover, the miRNA posttranscriptional control involves the presence of Smad binding elements (SBE) in the premature sequence of miRNAs, and we have not found any SBE in the corresponding sequences of the *mir-58* family.

Are there any examples of autoregulatory loops involving miRNAs and TGF-β? In mammals there are many cases of either miRNAs controlling TGF-β cell signalling or vice versa, and there are even some instances of autoregulatory loops running in both directions (64). However, as far as we know, no autoregulatory loop involving orthologs of *mir-58f* has been identified in any system. In Drosophila, Dpp/TGF-β positively modulates *bantam* expression through the Smad Mad, which together with Yorkie, bind directly to *bantam* and promote its transcription (33). And in humans, differentiation of smooth muscle cells is stimulated by TGF-β1's direct control over miR-143/145 transcription (65), which is a putative homologue of miR-58 (21). What our results suggest is that perhaps these other systems may conserve still undiscovered mechanisms of TGF-β regulation by the corresponding miR-58 orthologs.

What should be the functional consequences of a negative feedback loop between TGF-β Sma/Mab and miR-58 in *C. elegans*? Autoregulatory negative feedback loops contribute to biological homeostasis by limiting the range of outcoming gene expression within certain borders for a given genetic network (66). Thus, they are thought to favour stable responses (biochemical or phenotypic) to genetic or environmental alterations. On the other hand, it has been suggested that miRNAs, rather than promoting drastic variation in protein expression, buffer perturbations and confer robustness (67,68). It is possible that negative feedback loops are a way for miRNAs to acquire those properties. Therefore, in our system, the identified negative feedback loop might help to bring the TGF-β Sma/Mab signalling into a convenient equilibrium. And in doing so, the loop presumably helps nematodes to attain optimal body sizes or other fitting phenotypes, maybe also as part of a responding mechanism to the always-challenging environmental conditions (27,69,70).

It is interesting to compare such a suggested homeostatic process to the positive feedback loop that we discussed in relation to the TGF-β Dauer signalling pathway (Figure 10). This second kind of genetic loop, in contrast to the previous, enhances or amplifies the final molecular outcome, and for that reason it should ensure a particular physiological response. The decision whether to enter the dauer stage or not must be unambiguous. No in-between phenotype would be adaptive at all. Therefore, we would find the positive feedback loop at the TGF-β Dauer pathway rather useful for a developing *C. elegans* larva when having to take an unequivocal dauer decision.

## Supplementary Material

SUPPLEMENTARY DATA
