# Supplementary material for: miR-58 family and TGF-β pathways regulate each other in Caenorhabditis elegans
Source: Nucleic Acids Res. 2015 Sep 22;43(20):9978–93. doi: 10.1093/nar/gkv923 (PMC4783514; doi:10.1093/nar/gkv923)
Supplement: SUPPLEMENTARY DATA [file supp_43_20_9978__index.html]

miR-58 family and TGF-β pathways regulate each other in Caenorhabditis elegans — miR-58 family and TGF-β pathways regulate each other in Caenorhabditis elegans — SUPPLEMENTARY DATA 

# miR-58 family and TGF-β pathways regulate each other in *Caenorhabditis elegans*

## SUPPLEMENTARY DATA

- SUPPLEMENTARY DATA
