# Supplementary material for: miR-58 family and TGF-β pathways regulate each other in Caenorhabditis elegans
Source: Nucleic Acids Res. 2015 Sep 22;43(20):9978–93. doi: 10.1093/nar/gkv923 (PMC4783514; doi:10.1093/nar/gkv923)
Supplement: SUPPLEMENTARY DATA [file supp_gkv923_nar-02290-x-2015-File012.pdf]

SUPPORTING INFORMATION

miR-58 Family and TGF-β Pathways Regulate each other in *Caenorhabditis elegans*

María Pilar de Lucas[1], Alberto G. Sáez[1] and Encarnación Lozano[1*]

[1] Unidad Funcional de Investigación de Enfermedades Crónicas, Instituto de Salud Carlos III, 28220, Majadahonda, Madrid, Spain

* To whom correspondence should be addressed; Email: encarnilozano@gmail.com

Submitted to *NAR* (13 August 2015)

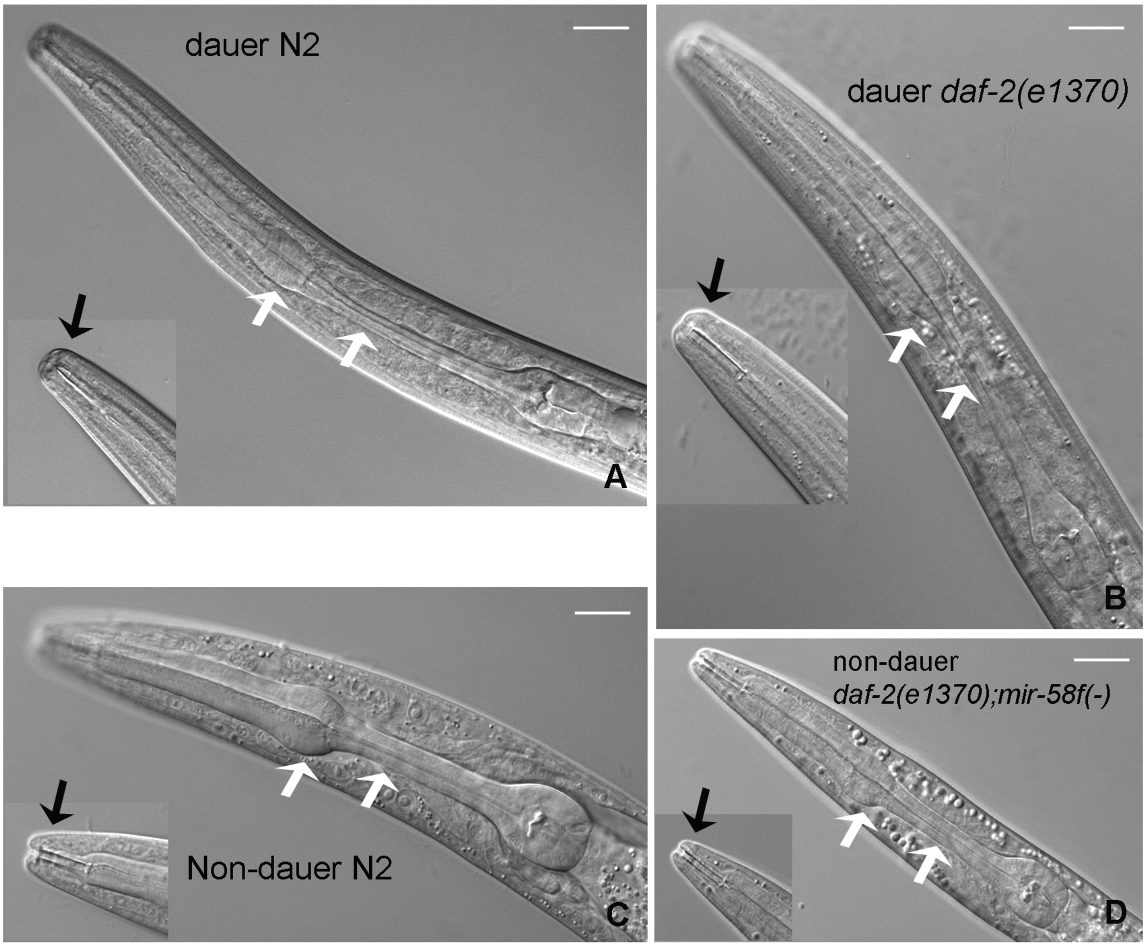

Figure S1

Figure S1. Downregulation of *daf-2* in *mir-58f(-)* mutants does not allow dauer formation. Worms were synchronized and cultured at 25.5ºC. Confocal images of dauer N2 **(A)**, dauer *daf-2(e1370)* **(B)**, L2 larvae of N2 **(C)** and *daf-2(e1370);mir-58f(-)* non-dauer arrested larvae **(D)** were taken with a 63x objective. Both N2 and *daf-2(e1370)* dauers **(A and B)** show constricted pharynges and closed mouths, whereas L2 N2 **(C)** and *mir-58f(-);daf-2(e1370)* **(D)** do not. Black arrows point to the details in mouth worms. White arrows point to the morphology of the pharynges. White bar represents 10 μm.

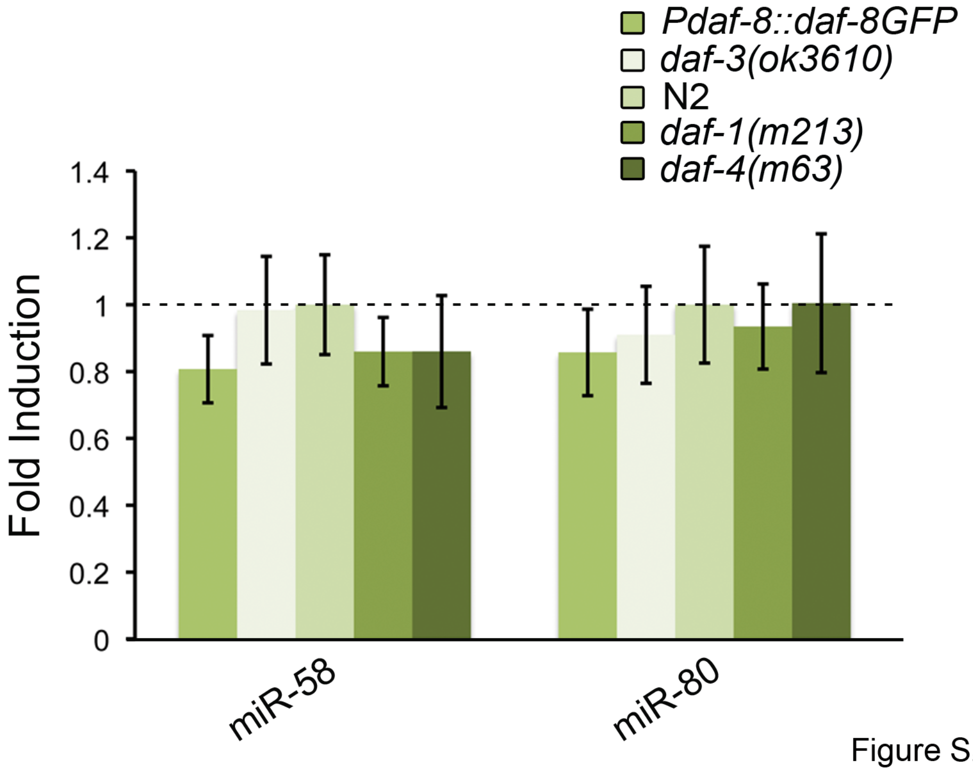

Figure S2

Figure S2. An altered TGF-β Dauer pathway does not reduce the transcriptional expression of *mir-58* family members in L1. qPCRs of mature miR-58 and miR-80 in *daf-8(++)*, *daf-3(ok3610)*, N2, *daf-1(m213)* and *daf-4(m63)* L1 worms are shown. Data represent the average of two independent experiments. Error bars indicate standard deviations.

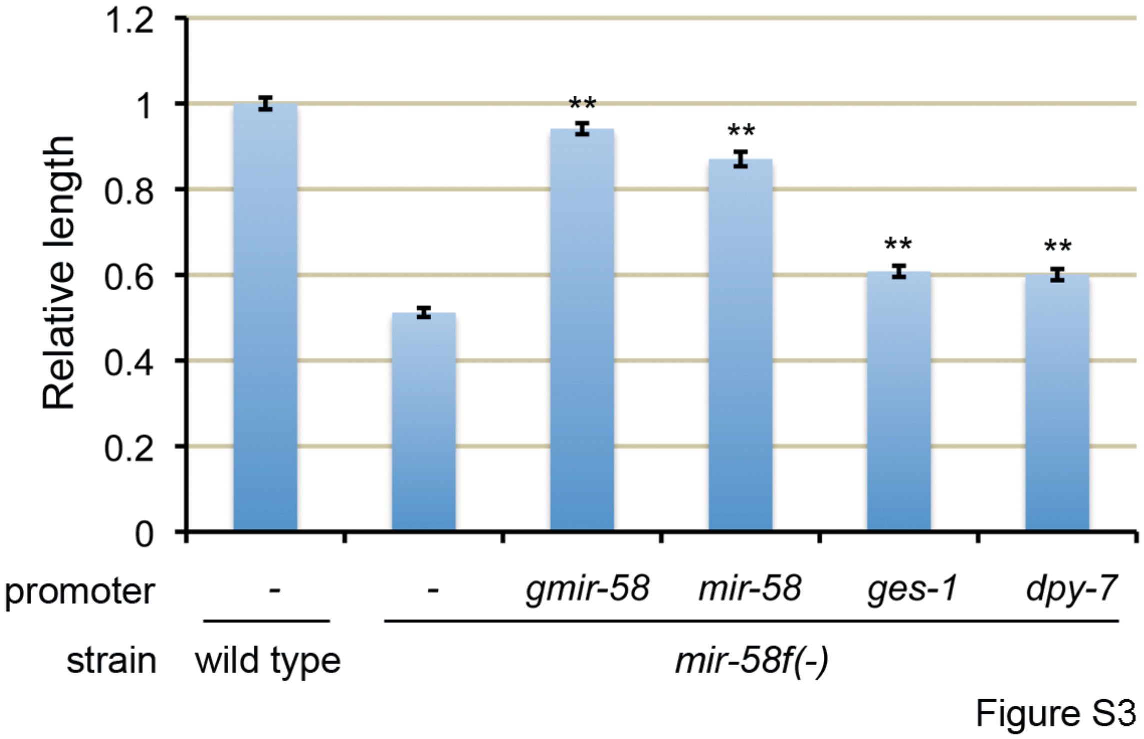

Figure S3. Targeted expression in gut or hypodermis of miR-58 partially recovers wild type body length. *mir-58* is expressed as genomic *mir-58* (*gmir-58*) or *pre-mir-58* with different tissue-specific promoters: *mir-58* own promoter (*mir-58*), intestinal (*ges-1*) and hypodermal (*dpy-7*). Data represent two independent experiments. Error bars indicate 95% confidence intervals. **p<0.001, referring to comparisons between N2 and each transgenic strain.

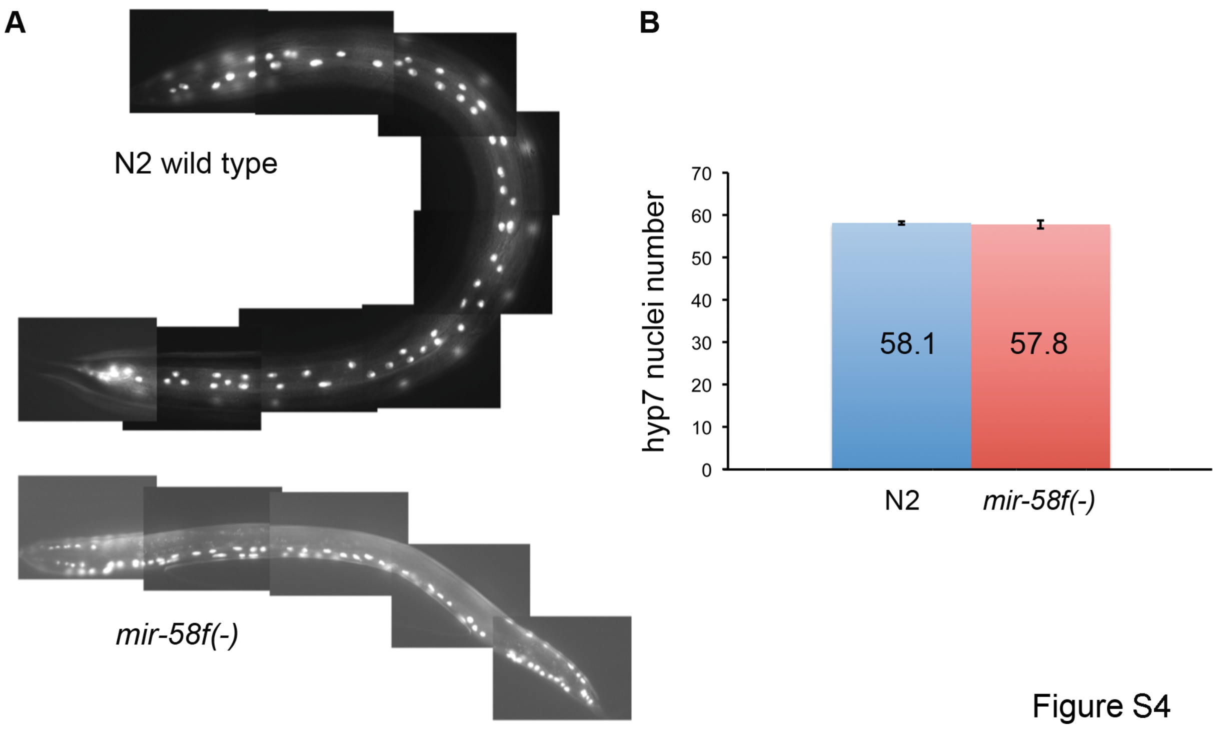

Figure S4

Figure S4. *mir-58* family mutant contains wild type hypodermal nuclei numbers. N2 and MT15563 *mir-58f(-)* were microinjected with a $P_{dpy\text{-}7}$*::4xNLS::gfp* construct to identify nuclei in the hypodermal tissue. $P_{dpy\text{-}7}$*::4xNLS::gfp* expresses GFP exclusively in hypodermal nuclei. Plates with adult worms were kept at 5ºC while their nuclei were counted under a stereomicroscope. **(A)** Composite microscope pictures show representative adults of N2 (upper) and *mir-58f(-)* (lower). **(B)** Quantification of nuclei in N2 and *mir-58(-)* adult worms. Error bars indicate 95% confidence intervals.

Table S1. New strains generated in this study

Strain	*Genotype*
ENL13	sma-6(wk7) II; madEx13 [(P_{sma-6}::genomic sma-6::3'UTR WT_{sma-6}) + pGK10 (P_{sca-1}::gfp::3'UTR_{unc-54}) + pHygroSfi]
ENL14	sma-6(wk7) II; madEx14 [(P_{sma-6}::genomic sma-6::3'UTR MUT_{sma-6}) + pGK10 (P_{sca-1}::gfp::3'UTR_{unc-54}) + pHygroSfi]
ENL16.1	mir-80(nDf53) III;mir-58(n4640) IV;mir-81&mir-82(nDf54) X; madEx13 [(P_{sma-6}::genomic sma-6::3'UTR WT_{sma-6}) + pGK10 (P_{sca-1}::gfp::3'UTR_{unc-54}) + pHygroSfi]
ENL16.2	mir-80(nDf53) III;mir-58(n4640) IV;mir-81&mir-82(nDf54) X; madEx13 [(P_{sma-6}::genomic sma-6::3'UTR WT_{sma-6}) + pGK10 (P_{sca-1}::gfp::3'UTR_{unc-54}) + pHygroSfi]
ENL16.3	mir-80(nDf53) III;mir-58(n4640) IV;mir-81&mir-82(nDf54) X; madEx13 [(P_{sma-6}::genomic sma-6::3'UTR WT_{sma-6}) + pGK10 (P_{sca-1}::gfp::3'UTR_{unc-54}) + pHygroSfi]
ENL17.1	mir-80(nDf53) III;mir-58(n4640) IV;mir-81&mir-82(nDf54) X; madEx14 [(P_{sma-6}::genomic sma-6::3'UTR MUT_{sma-6}) + pGK10 (P_{sca-1}::gfp::3'UTR_{unc-54}) + pHygroSfi]
ENL17.2	mir-80(nDf53) III;mir-58(n4640) IV;mir-81&mir-82(nDf54) X; madEx14 [(P_{sma-6}::genomic sma-6::3'UTR MUT_{sma-6}) + pGK10 (P_{sca-1}::gfp::3'UTR_{unc-54}) + pHygroSfi]
ENL19	mir-58(n4640) IV; ctIs40 X [ZC421(dbl-1(+)) + pTG98(sur-5::gfp)]
ENL20	mir-80(nDf53) III;daf-1(m213) mir-58(n4640) IV;mir-81&mir-82(nDf54) X
ENL21	daf-2(e1370) mir-80(nDf53) III;mir-58(n4640) IV;mir-81&mir-82(nDf54) X
ENL22	madEx16 [Pdpy-7::4xNLS::gfp::3'UTR_{unc-54} + p374 (P_{trx-3}::mCherry::3'UTR_{unc-54}) + pHygroSfi]
ENL23	mir-80(nDf53) III;mir-58(n4640) IV;mir-81&mir-82(nDf54) X; madEx16 [P_{dpy-7}::4xNLS::gfp::3'UTR_{unc-54} + p374 (P_{trx-3}::mCherry::3'UTR_{unc-54}) + pHygroSfi]
ENL24.1	madEx17 [P_{sma-6}::mCherry::3'$UTR_{sma-6WT}$ + pGK10 (P_{sca-1}::gfp::3'UTR_{unc-54}) + pHygroSfi]
ENL24.2	madEx17 [P_{sma-6}::mCherry::3'$UTR_{sma-6WT}$ + pGK10 (P_{sca-1}::gfp::3'UTR_{unc-54}) + pHygroSfi]
ENL25.1	mir-80(nDf53) III;mir-58(n4640) IV;mir-81&mir-82(nDf54) X; madEx17 [P_{sma-6}::mCherry::3'$UTR_{sma-6WT}$ + pGK10 (P_{sca-1}::gfp::3'UTR_{unc-54}) + pHygroSfi]
ENL25.2	mir-80(nDf53) III;mir-58(n4640) IV;mir-81&mir-82(nDf54) X; madEx17 [P_{sma-6}::mCherry::3'$UTR_{sma-6WT}$ + pGK10 (P_{sca-1}::gfp::3'UTR_{unc-54}) + pHygroSfi]
ENL26	madEx18 [P_{sma-6}::mCherry::3'$UTR_{sma-6MUT}$ + pGK10 (P_{sca-1}::gfp::3'UTR_{unc-54}) + pHygroSfi]
ENL27	mir-80(nDf53) III;mir-58(n4640) IV;mir-81&mir-82(nDf54) X; madEx18 [P_{sma-6}::mCherry::3'$UTR_{sma-6MUT}$ + pGK10 (P_{sca-1}::gfp::3'UTR_{unc-54}) + pHygroSfi]
ENL28.1	madEx19 [P_{daf-4}::mCherry::3'$UTR_{sma-6WT}$ + pGK10 (P_{sca-1}::gfp::3'UTR_{unc-54}) + pHygroSfi]

ENL28.2	madEx19 [P_{daf-4}::mCherry::3'UTR$_{sma-6WT}$ + pGK10 (P_{sca-1}::gfp::3'UTR$_{unc-54}$) + pHygroSfi]
ENL28.3	madEx19 [P_{daf-4}::mCherry::3'UTR$_{sma-6WT}$ + pGK10 (P_{sca-1}::gfp::3'UTR$_{unc-54}$) + pHygroSfi]
ENL28.4	madEx19 [P_{daf-4}::mCherry::3'UTR$_{sma-6WT}$ + pGK10 (P_{sca-1}::gfp::3'UTR$_{unc-54}$) + pHygroSfi]
ENL29.1	mir-80(nDf53) III;mir-58(n4640) IV;mir-81&mir-82(nDf54) X; madEx19 [P_{daf-4}::mCherry::3'UTR$_{sma-6WT}$ + pGK10 (P_{sca-1}::gfp::3'UTR$_{unc-54}$) + pHygroSfi]
ENL29.2	mir-80(nDf53) III;mir-58(n4640) IV;mir-81&mir-82(nDf54) X; madEx19 [P_{daf-4}::mCherry::3'UTR$_{sma-6WT}$ + pGK10 (P_{sca-1}::gfp::3'UTR$_{unc-54}$) + pHygroSfi]
ENL30.1	madEx20 [P_{daf-4}::mCherry::3'UTR$_{sma-6MUT}$ + pGK10 (P_{sca-1}::gfp::3'UTR$_{unc-54}$) + pHygroSfi]
ENL30.2	madEx20 [P_{daf-4}::mCherry::3'UTR$_{sma-6MUT}$ + pGK10 (P_{sca-1}::gfp::3'UTR$_{unc-54}$) + pHygroSfi]
ENL30.3	madEx20 [P_{daf-4}::mCherry::3'UTR$_{sma-6MUT}$ + pGK10 (P_{sca-1}::gfp::3'UTR$_{unc-54}$) + pHygroSfi]
ENL30.4	madEx20 [P_{daf-4}::mCherry::3'UTR$_{sma-6MUT}$ + pGK10 (P_{sca-1}::gfp::3'UTR$_{unc-54}$) + pHygroSfi]
ENL31.1	mir-80(nDf53) III;mir-58(n4640) IV;mir-81&mir-82(nDf54) X; madEx20 [P_{daf-4}::mCherry::3'UTR$_{sma-6MUT}$ + pGK10 (P_{sca-1}::gfp::3'UTR$_{unc-54}$) + pHygroSfi]
ENL31.2	mir-80(nDf53) III;mir-58(n4640) IV;mir-81&mir-82(nDf54) X; madEx20 [P_{daf-4}::mCherry::3'UTR$_{sma-6MUT}$ + pGK10 (P_{sca-1}::gfp::3'UTR$_{unc-54}$) + pHygroSfi]
ENL31.3	mir-80(nDf53) III;mir-58(n4640) IV;mir-81&mir-82(nDf54) X; madEx20 [P_{daf-4}::mCherry::3'UTR$_{sma-6MUT}$ + pGK10 (P_{sca-1}::gfp::3'UTR$_{unc-54}$) + pHygroSfi]
ENL31.4	mir-80(nDf53) III;mir-58(n4640) IV;mir-81&mir-82(nDf54) X; madEx20 [P_{daf-4}::mCherry::3'UTR$_{sma-6MUT}$ + pGK10 (P_{sca-1}::gfp::3'UTR$_{unc-54}$) + pHygroSfi]
ENL32.1	madEx21 [P_{daf-4}::mCherry::3'UTR$_{daf-4WT}$ + pGK10 (P_{sca-1}::gfp::3'UTR$_{unc-54}$) + pHygroSfi]
ENL32.2	madEx21 [P_{daf-4}::mCherry::3'UTR$_{daf-4WT}$ + pGK10 (P_{sca-1}::gfp::3'UTR$_{unc-54}$) + pHygroSfi]
ENL32.3	madEx21 [P_{daf-4}::mCherry::3'UTR$_{daf-4WT}$ + pGK10 (P_{sca-1}::gfp::3'UTR$_{unc-54}$) + pHygroSfi]
ENL33.1	mir-80(nDf53) III;mir-58(n4640) IV;mir-81&mir-82(nDf54) X; madEx21 [P_{daf-4}::mCherry::3'UTR$_{daf-4WT}$ + pGK10 (P_{sca-1}::gfp::3'UTR$_{unc-54}$) + pHygroSfi]
ENL33.2	mir-80(nDf53) III;mir-58(n4640) IV;mir-81&mir-82(nDf54) X; madEx21 [P_{daf-4}::mCherry::3'UTR$_{daf-4WT}$ + pGK10 (P_{sca-1}::gfp::3'UTR$_{unc-54}$) + pHygroSfi]
ENL33.3	mir-80(nDf53) III;mir-58(n4640) IV;mir-81&mir-82(nDf54) X; madEx21 [P_{daf-4}::mCherry::3'UTR$_{daf-4WT}$ + pGK10 (P_{sca-1}::gfp::3'UTR$_{unc-54}$) + pHygroSfi]
ENL34.1	madEx22 [P_{daf-4}::mCherry::3'UTR$_{daf-4MUT}$ + pGK10 (P_{sca-1}::gfp::3'UTR$_{unc-54}$) + pHygroSfi]

ENL34.2	*madEx22 [P_{daf-4}::mCherry::3'UTR$_{daf-4MUT}$ + pGK10 (P_{sca-1}::gfp::3'UTR$_{unc-54}$) + pHygroSfi]*
ENL35.1	*mir-80(nDf53) III;mir-58(n4640) IV;mir-81&mir-82(nDf54) X; madEx22 [P_{daf-4}::mCherry::3'UTR$_{daf-4MUT}$ + pGK10 (P_{sca-1}::gfp::3'UTR$_{unc-54}$) + pHygroSfi]*
ENL35.2	*mir-80(nDf53) III;mir-58(n4640) IV;mir-81&mir-82(nDf54) X; madEx22 [P_{daf-4}::mCherry::3'UTR$_{daf-4MUT}$ + pGK10 (P_{sca-1}::gfp::3'UTR$_{unc-54}$) + pHygroSfi]*
ENL36.1	*madEx23 [P_{dbl-1}::mCherry::3'UTR$_{dbl-1WT}$ + pGK10 (P_{sca-1}::gfp::3'UTR$_{unc-54}$) + pHygroSfi]*
ENL36.2	*madEx23 [*P_{dbl-1}::mCherry::3'UTR$_{dbl-1WT}$ *+ pGK10 (P_{sca-1}::gfp::3'UTR$_{unc-54}$) + pHygroSfi]*
ENL37.1	*mir-80(nDf53) III;mir-58(n4640) IV;mir-81&mir-82(nDf54) X; madEx23 [P_{dbl-1}::mCherry::3'UTR$_{dbl-1WT}$ + pGK10 (P_{sca-1}::gfp::3'UTR$_{unc-54}$) + pHygroSfi]*
ENL37.2	*mir-80(nDf53) III;mir-58(n4640) IV;mir-81&mir-82(nDf54) X; madEx23 [P_{dbl-1}::mCherry::3'UTR$_{dbl-1WT}$ + pGK10 (P_{sca-1}::gfp::3'UTR$_{unc-54}$) + pHygroSfi]*
ENL38	*madEx24 [P_{dbl-1}::mCherry::3'UTR$_{dbl-1MUT}$ + pGK10 (P_{sca-1}::gfp::3'UTR$_{unc-54}$) + pHygroSfi]*
ENL39	*mir-80(nDf53) III;mir-58(n4640) IV;mir-81&mir-82(nDf54) X; madEx24 [P_{dbl-1}::mCherry::3'UTR$_{dbl-1MUT}$ + pGK10 (P_{sca-1}::gfp::3'UTR$_{unc-54}$) + pHygroSfi]*
ENL40.1	*madEx25 [P_{daf-1}::mCherry::3'UTR$_{daf-1WT}$ + pGK10 (P_{sca-1}::gfp::3'UTR$_{unc-54}$) + pHygroSfi]*
ENL40.2	*madEx25 [P_{daf-1}::mCherry::3'UTR$_{daf-1WT}$ + pGK10 (P_{sca-1}::gfp::3'UTR$_{unc-54}$) + pHygroSfi]*
ENL41.1	*mir-80(nDf53) III;mir-58(n4640) IV;mir-81&mir-82(nDf54) X; madEx25 [P_{daf-1}::mCherry::3'UTR$_{daf-1WT}$ + pGK10 (P_{sca-1}::gfp::3'UTR$_{unc-54}$) + pHygroSfi]*
ENL41.2	*mir-80(nDf53) III;mir-58(n4640) IV;mir-81&mir-82(nDf54) X; madEx25 [P_{daf-1}::mCherry::3'UTR$_{daf-1WT}$ + pGK10 (P_{sca-1}::gfp::3'UTR$_{unc-54}$) + pHygroSfi]*
ENL42.1	*madEx26 [P_{daf-1}::mCherry::3'UTR$_{daf-1MUT}$ + pGK10 (P_{sca-1}::gfp::3'UTR$_{unc-54}$) + pHygroSfi]*
ENL42.2	*madEx26 [P_{daf-1}::mCherry::3'UTR$_{daf-1MUT}$ + pGK10 (P_{sca-1}::gfp::3'UTR$_{unc-54}$) + pHygroSfi]*
ENL43.1	*mir-80(nDf53) III;mir-58(n4640) IV;mir-81&mir-82(nDf54) X; madEx26 [P_{daf-1}::mCherry::3'UTR$_{daf-1MUT}$ + pGK10 (P_{sca-1}::gfp::3'UTR$_{unc-54}$) + pHygroSfi]*
ENL43.2	*mir-80(nDf53) III;mir-58(n4640) IV;mir-81&mir-82(nDf54) X; madEx26 [P_{daf-1}::mCherry::3'UTR$_{daf-1MUT}$ + pGK10 (P_{sca-1}::gfp::3'UTR$_{unc-54}$) + pHygroSfi]*
ENL44.1	*madEx27 [P_{sma-6}::mCherry::3'UTR$_{unc-54}$ + pGK10 (P_{sca-1}::gfp::3'UTR$_{unc-54}$) + pHygroSfi]*
ENL44.2	*madEx27 [P_{sma-6}::mCherry::3'UTR$_{unc-54}$ + pGK10 (P_{sca-1}::gfp::3'UTR$_{unc-54}$) + pHygroSfi]*

ENL45.1	*mir-80(nDf53) III;mir-58(n4640) IV;mir-81&mir-82(nDf54) X; madEx27 [*P_{sma-6}*::mCherry::3'*UTR_{unc-54} *+ pGK10 (*P_{sca-1}*::gfp::3'*UTR_{unc-54}*) + pHygroSfi]*
ENL45.2	*mir-80(nDf53) III;mir-58(n4640) IV;mir-81&mir-82(nDf54) X; madEx27 [*P_{sma-6}*::mCherry::3'*UTR_{unc-54} *+ pGK10 (*P_{sca-1}*::gfp::3'*UTR_{unc-54}*) + pHygroSfi]*
ENL46.1	*dbl-1(nk3) V; madEx27 [*P_{sma-6}*::mCherry::3'*UTR_{unc-54} *+ pGK10 (*P_{sca-1}*::gfp::3'*UTR_{unc-54}*) + pHygroSfi]*
ENL46.2	*dbl-1(nk3) V; madEx27 [*P_{sma-6}*::mCherry::3'*UTR_{unc-54} *+ pGK10 (*P_{sca-1}*::gfp::3'*UTR_{unc-54}*) + pHygroSfi]*
ENL47.1	*ctIs40 X [ZC421(dbl-1(+)) + pTG98(sur-5::gfp)]; madEx27 [*P_{sma-6}*::mCherry::3'*UTR_{unc-54} *+ pGK10 (*P_{sca-1}*::gfp::3'*UTR_{unc-54}*) + pHygroSfi]*
ENL47.2	*ctIs40 X [ZC421(dbl-1(+)) + pTG98(sur-5::gfp)]; madEx27 [*P_{sma-6}*::mCherry::3'*UTR_{unc-54} *+ pGK10 (*P_{sca-1}*::gfp::3'*UTR_{unc-54}*) + pHygroSfi]*
ENL76	EUB0032 *(*P_{mir-58}*::gfp); dbl-1(nk3) V*
ENL77	EUB0032 *(*P_{mir-58}*::gfp); sma-10(ok2224) IV*
ENL78	EUB0032 *(*P_{mir-58}*::gfp); daf-4(m63) III*
ENL79	EUB0032 *(*P_{mir-58}*::gfp); madEx28 [WRM0624CB02; p374 (*P_{trx-3}*::mCherry::3'*UTR_{unc-54}*); pHygroSfi]*
ENL80	EUB0032 *(*P_{mir-58}*::gfp); madEx29 [p374 (*P_{trx-3}*::mCherry::3'*UTR_{unc-54}*) + pHygroSfi]*
ENL81.1	*madEx30 [pGK10 (*P_{sca-1}*::gfp::3'*UTR_{unc-54}*) + pHygroSfi]*
ENL81.2	*madEx30 [pGK10 (*P_{sca-1}*::gfp::3'*UTR_{unc-54}*) + pHygroSfi]*
ENL82.1	*sma-6(wk7) II; madEx30 [pGK10 (*P_{sca-1}*::gfp::3'*UTR_{unc-54}*) + pHygroSfi]*
ENL82.2	*sma-6(wk7) II; madEx30 [pGK10 (*P_{sca-1}*::gfp::3'*UTR_{unc-54}*) + pHygroSfi]*
ENL83.1	*daf-1(m213) IV; madEx30 [pGK10 (*P_{sca-1}*::gfp::3'*UTR_{unc-54}*) + pHygroSfi]*
ENL83.2	*daf-1(m213) IV; madEx30 [pGK10 (*P_{sca-1}*::gfp::3'*UTR_{unc-54}*) + pHygroSfi]*
ENL85.1	*mir-80(nDf53) III; mir-58(n4640) IV; mir-81&mir-82(nDf54) X; madEx30 [pGK10 (*P_{sca-1}*::gfp::3'*UTR_{unc-54}*) + pHygroSfi]*
ENL85.2	*mir-80(nDf53) III; mir-58(n4640) IV; mir-81&mir-82(nDf54) X; madEx30 [pGK10 (*P_{sca-1}*::gfp::3'*UTR_{unc-54}*) + pHygroSfi]*
ENL88	EUB0032 *(*P_{mir-58}*::gfp); daf-3(ok3610) X*
ENL89	EUB0032 *(*P_{mir-58}*::gfp); daf-1(m213) IV*
ENL90	*mir-80(nDf53) III; mir-58(n4640) IV*
ENL91	*mir-80(nDf53) III; mir-81&mir-82(nDf54) X*
ENL92	*mir-58(n4640) IV; mir-81&mir-82(nDf54) X*
ENL93	*mir-58(n4640) IV; dbl-1(nk3) V*
ENL105	*mir-80(nDf53) III; mir-58(n4640) IV; mir-81&mir-82(nDf54) X; madEx35 [pCR2.1 (mir-58++) + pGK10 (*P_{sca-1}*::gfp::3'*UTR_{unc-54}*) + pHygroSfi]*
ENL107	*mir-80(nDf53) III; mir-58(n4640) IV; mir-81&mir-82(nDf54) X; madEx37 [*P_{mir-58}*::premir-58) + pGK10 (*P_{sca-1}*::gfp::3'*UTR_{unc-54}*) + pHygroSfi]*
ENL110	*mir-80(nDf53) III; mir-58(n4640) IV; mir-81&mir-82(nDf54) X; madEx40 [*P_{ges-1}*::premir-58) + pGK10 (*P_{sca-1}*::gfp::3'*UTR_{unc-54}*) + pHygroSfi]*
ENL111	*mir-80(nDf53) III; mir-58(n4640) IV; mir-81&mir-82(nDf54) X; madEx41 [*P_{dpy-7}*::premir-58) + pGK10 (*P_{sca-1}*::gfp::3'*UTR_{unc-54}*) + pHygroSfi*

Table S2. Primers used for double/triple miRNA mutant screen, hyp-7 quantitation and transgenes

**Genotype**	**Primers**	**Sequence**
mir-58(n4640)	MT15024F2	GTACACCGCTCTTGC
	MT15024R3	AGATCAAGACCGTACTC
	MT15024R4	CAGTTGTCCTTCAATCG
mir-80(nDf53)	MT13949F	TGGGCAACAAACTCATTTCG
	MT13949R1	ATTCTGAACAATCCGCAAGC
	MT13949R2	AATCCGCTTCCAATCGTTCG
mir-81&mir-82(nDf54)	MT13954A	GGTATATGTCCACTCTCTCGT
	MT13954B	TCGCTGCAATCCGAAGATGG
	MT13954C	CAAATTGACGGTAGAGTGGCG
dbl-1(nk3)	MS7	CATGGACAAACATCGGGGA
	MS11	CGTGTACACAAATCTGTTCG
	T25F10.2F	CGACTCTGTGCGGACAACTA
	T25F10.2R	AAGCATCGTAGCCCTCTGAA
daf-1(m213)	DAF-1F	CGGAGATCTTTGAGACGCGGATG
	DAF-1R	CTCGGTCCACAATAGGAGCC
dpy-7 promoter	DPY-7F	GAAATGGTGAGTGTTCGAGCTC
	DPY-7R	TTATCTGGAACAAAATGTAAGAATATTC
ges-1 promoter	Pges-1F	TTGGCATGAATACAGTGAACACAG
	Pges-1R	GAATTCAAAGATAAGATATGTAATAGAT TTTTGAAGC
dbl-1 promoter	Pdbl-1F3	TTCATCCATTCAACTGTCCATTC
	Pdbl-1R3	AGTTGAGTTGGGCGCATCAGGC
sma-6 promoter	Psma-6F	CTTCACTGCAGCAACG
	Psma-6R	TAAATCTGAAATTTGCAAATAAATTG
daf-4 promoter	Pdaf-4F	AAGTAGGCAATCGGCAATAATGG
	Pdaf-4R	CTGAAAATTGAAGGTAGTTGAATTATTG
daf-1 promoter	Pdaf-1F	AAACTGTGGCCTAGTTCTGC
	Pdaf-1R	AATTTATTTCCCAAGAGTTTAGTTCATC
mir-58 promoter	Pmir-58F	TTAAGGTAATACCGAATTTTGGCTC
	Pmir-58R	ATCGGGGGATAGGTCGAGAGG
mir-58 genomic	mir-58OEF	TTAAGGTAATACCGAATTTTGGCTC
	mir-58OEFR5	CCCAGGTAAGTCTTCAAGTGC
pre-mir-58	premir-58F	GTTCGTCATATCCATTGCCCTAC
	premir-58R	ACTCTAGCTCAGTCCATTGCCG

Table S3. Primers used for 3'UTR wild type and mutant generation[a]

3'UTR (size)	Primers	Sequence
dbl-1 (470nt)	dbl-1UTRF	gtacccgcttctatgtcgc
	dbl-1UTRR	tcaaataccaaaatgtt
	dbl-1UTRmutF	atatttgatGAGatagtttttgtgtc
	dbl-1UTRmutR	gacacaaaaactatCTCatcaaatat
sma-6 (216nt)	sma-6UTRF	cccactttctgatcttac
	sma-6UTRR	tacaggtctggaacaat
	sma-6UTRmutF	ttaaagcgatGAGtctaataatttcc
	sma-6UTRmutR	ggaaattattagaCTCatcgctttaa
daf-1 (455nt)	daf-1UTRF	agcccattttatcttccgttc
	daf-1UTRR	aaattttttattttcattttc
	daf-1MUT1F	cccccatatcgatgatGAGatttctcttcc
	daf-1MUT1R	ggaagagaaatCTCatcatcgatatggggg
	daf-1MUT2F	ctgtaccatgatGAGaagtgtttttg
	daf-1MUT2R	caaaaacacttCTCatcatggtacag
daf-4 (476nt)	daf-4UTRF	ctattcattcaaatctaacatt
	daf-4UTRR	aagaaacattatttaattttaa
	daf-4MUT1F	cccctaatgatGAGaatttgagcacaacc
	daf-4MUT1R	ggttgtgctcaaattCTCatcattagggg
	daf-4MUT2F	ctcttccaaaatctctgatGAGttgcattgtctc
	daf-4MUT2R	gagacaatgcaaCTCatcagagattttggaagag
	daf-4MUT3F	cattcactatattcattgatGAGtgataactttgtattgg
	daf-4MUT3R	ccaatacaaagttatcaCTCatcaatgaatatagtgaatg
daf-7 (489nt)	daf-7UTRF	attccacttccgattgtcac
	daf-7UTRR	ataatttattcctcaatcacagtg
	daf-7MUT1F	gaatcaaaattcatgatGAGaaccccgtagtc
	daf-7MUT1R	gactacggggttCTCatcatgaattttgattc
	daf-7MUT23F	ctgtcggtGAGctttccctcgatGAGatcccag
	daf-7MUT23R	ctgggatCTCatcgagggaaagCTCaccgacag

[a] Uppercase nucleotides correspond to the mutated sites

Table S4. Plasmid concentrations (ng/μl) for transgenic arrays[a]

Transgene plasmid name	Plasmid	pHygroSfi	p374	pGK10
$P_{sma\text{-}6}$::mCherry::3'$UTR_{sma\text{-}6WT}$	50	50	-	20
$P_{sma\text{-}6}$::mCherry::3'$UTR_{sma\text{-}6MUT}$	50	50	-	20
$P_{daf\text{-}1}$::mCherry::3'$UTR_{daf\text{-}1WT}$	60	30	-	20
$P_{daf\text{-}1}$::mCherry::3'$UTR_{daf\text{-}1MUT}$	60	30	-	20
$P_{daf\text{-}4}$::mCherry::3'$UTR_{daf\text{-}4WT}$	50	30	-	20
$P_{daf\text{-}4}$::mCherry::3'$UTR_{daf\text{-}4MUT}$	50	30	-	20
$P_{daf\text{-}4}$::mCherry::3'$UTR_{sma\text{-}6WT}$	50	30	-	20
$P_{daf\text{-}4}$::mCherry::3'$UTR_{sma\text{-}6MUT}$	50	30	-	20
$P_{dbl\text{-}1}$::mCherry::3'$UTR_{dbl\text{-}1WT}$	60/30	40/50	-	20
$P_{dbl\text{-}1}$::mCherry::3'$UTR_{dbl\text{-}1MUT}$	60	30	-	20
$P_{dpy\text{-}7}$::4xNLS::gfp::3'$UTR_{unc\text{-}54}$	50	50	30	-
WRM0624CB02 *dbl-1* fosmid	50	30	40	-
$P_{sma\text{-}6}$::mCherry::3'$UTR_{unc\text{-}54}$	50	50	-	20
pCR2.1-mir-58	20	50	-	20
$P_{ges\text{-}1}$::pre-mir-58	20	50	-	20
$P_{dpy\text{-}7}$::pre-mir-58	20	50	-	20
$P_{mir\text{-}58}$::pre-mir-58	20	50	-	20
$P_{sma\text{-}6}$::sma-6::3'$UTR_{sma\text{-}6WT}$	20	30	-	20
$P_{sma\text{-}6}$::sma-6::3'$UTR_{sma\text{-}6MUT}$	20	30	-	20
Control mCherry	-	30	50	-
Control gfp	-	30	-	20

[a] Plasmids were mixed with 80 ng/μl 1kb DNA ladder (Invitrogen)

Table S5. Predicted binding positions of mir-58 family members to *dbl-1*, *sma-6*, *daf-4*, *daf-1* and *daf-7* 3'UTRs

```
dbl-1 3'UTR (392 - 413)    5'  AAAUUCAAAAUAUU---UGAUCUCA 3'
                                 |||     ||:|   :|||||||
miR-58                     3'    UAA-CGGCAUGACUUGCUAGAGU 5'

dbl-1 3'UTR (392 - 413)    5'     AAAUUCAAAAUAUUUGAUCUCA 3'
                                     ||| || ||  ||||||||
miR-80                     3'  AGCCGAAAG-UUGAU-UACUAGAGU 5'

dbl-1 3'UTR (392 - 413)    5'  A--A--AUUCAAAAU-AUUUGAUCUCA 3'
                               |  |   |||   |: |  ||||||||
miR-81                     3'  UGAUCGAAAG---UGCU--ACUAGAGU 5'

dbl-1 3'UTR (392 - 413)    5'  AAAUU--CAAAAUAUU-----UGAUCUCA 3'
                                 |:|  |    |  ||     ||||||||
miR-82                     3'  --UGACCG----A-AAGUGCUACUAGAGU 5'

dbl-1 3'UTR (392 - 413)    5'    AAAU-UCAAAAUAUUUGAUCUCA 3'
                                   || ||  |||  ||||||||
miR-1834                   3'  AACCUAGAG--UUACCAACUAGAGA 5'


sma-6 3'UTR (1 – 17)       5'       CCCACUUUCUGAUCUUA 3'
                                    |  |||   :|||||:|
miR-58                     3'  UAACGGCAUGACUUGCUAGAGU 5'

sma-6 3'UTR (1 – 17)       5'        CCCACUUUCUGAUCUUA 3'
                                       || :|  ||||||:|
miR-80                     3'  AGCCGAAAGUUGAUUACUAGAGU 5'

sma-6 3'UTR (1 – 17)       5'   CCCACUUUC----UGAUCUUA 3'
                                |   |||||    ||||||:|
miR-81                     3'  UGAUCGAAAGUGCUACUAGAGU 5'

sma-6 3'UTR (1 – 17)       5'   CCCACUUUC----UGAUCUUA 3'
                                |   |||||    ||||||:|
miR-82                     3'  UGACCGAAAGUGCUACUAGAGU 5'

sma-6 3'UTR (1 – 17)       5'        CCCACUUUCUGAUCUUA 3'
                                     | || |   ||||||:
miR-1834                   3'  AACCUAGAGUUACCAACUAGAGA 5'
```

```
sma-6 3'UTR (93 – 114)      5'  G-U-CAAAAU-UUAAAGCGAUCUCU 3'
                                : | |    | :|  |:|||||||
miR-58                      3'  UAACG--GCAUGA-CUUGCUAGAGU 5'

sma-6 3'UTR (93 – 114)      5'  GUCAAAAUUUAA--AGCGAUCUCU 3'
                                 ||    ||:||  |: ||||||
miR-80                      3'   AGCCGAAAGUUGAUUACUAGAGU 5'

sma-6 3'UTR (93 – 114)      5'  GUCAAAAUUU-AAAGCGAUCUCU 3'
                                  | |  ||| |  : ||||||
miR-81                      3'   UGAUCGAAAGUGCUACUAGAGU 5'

sma-6 3'UTR (93 – 114)      5'  GUCAAAAUUU-AAAGCGAUCUCU 3'
                                  |    ||| |  : ||||||
miR-82                      3'   UGACCGAAAGUGCUACUAGAGU 5'

sma-6 3'UTR (93 – 114)      5'  GUCAAAAUUUAAAG--CGAUCUCU 3'
                                 |   | :|:|| |   |||||||
miR-1834                    3'   AACCUAGAGUUACCAACUAGAGA 5'


daf-4 3'UTR (127 – 148)     5'   UACGCGUCCCCUAAUGAUCUCA 3'
                                 |  ||||  |  ||:|||||||
miR-58                      3'  UAACGGCA-UGACUUGCUAGAGU 5'

daf-4 3'UTR (127 – 148)     5'  UAC-GC-GUCCCCUAAUGAUCUCA 3'
                                | | ||  ||  ||||||||||||
miR-80                      3'  A-GCCGAAAGUUGAUUACUAGAGU 5'

daf-4 3'UTR (127 – 148)     5'  UAC--GCGUCCCCUAAUGAUCUCA 3'
                                 ||  || | | |  |||||||||
miR-81                      3'   UGAUCGAAAGUG-CUACUAGAGU 5'

daf-4 3'UTR (127 – 148)     5'   UAC–GCGUCCCCUAAUGAUCUCA 3'
                                 ||  || | | |  |||||||||
miR-82                      3'   UGACCGAAAGUG-CUACUAGAGU 5'

daf-4 3'UTR (127 – 148)     5'  UACGCG-UCCCCU-AA----UGAUCUCA 3'
                                |  | | |   ||  ||    |||||||
miR-1834                    3'  A-AC-CUA---GAGUUACCAACUAGAGA 5'
```

```
daf-4 3’UTR (234 – 255)    5'    UCUUCCAAAAU-CU--CUGAUCUCU 3’
                                | | ||    | ||   :||||||
miR-58                     3'   UA-ACGG---CAUGACUUGCUAGAGU 5’

daf-4 3’UTR (234 – 255)    5'   U---CUUCCAAAAUCU-CUGAUCUCU 3’
                               |   ||| |  || ||  |||||||
miR-80                     3'   AGCCGAAAG--UU-GAUUACUAGAGU 5’

daf-4 3’UTR (234 – 255)    5'     U--CUUCCAAAAUCUCUGAUCUCU 3’
                                 |  ||| |   | |  |||||||
miR-81                     3'   UGAUCGAAAG---U-GCUACUAGAGU 5’

daf-4 3’UTR (234 – 255)    5'     U--CUUCCAAAAUCUCUGAUCUCU 3’
                                 |  ||| |   | |  |||||||
miR-82                     3'   UGACCGAAAG---U-GCUACUAGAGU 5’

daf-4 3’UTR (234 – 255)    5'   UCU---UC-CAAAAU-CUCUGAUCUCU 3’
                               | |   || |  |||  | ||||||||
miR-1834                   3'   A-ACCUAGAG--UUACCA-ACUAGAGA 5’


daf-4 3’UTR (351 – 372)    5'    UUCACUAUAUUCAUUGAUCUGU 5’
                                ||  |: ||:| | :||||| :
miR-58                     3'   UAA-CGGCAUGACUUGCUAGA-GU 3’

daf-4 3’UTR (351 – 372)    5'   UUCACUAUAUU--CAU--UGAUCUGU 5’
                                   |  : ||    |  |||||| :
miR-80                     3'      AGCCGAAAGUUGAUUACUAGA-GU 3

daf-4 3’UTR (351 – 372)    5'   UUCACUA--UAUUCAU--UGAUCUGU 5’
                                  ||||  | ||||:  |||||| :
miR-81                     3'      UGAUCGA-AAGUGCUACUAGA-GU 3’

daf-4 3’UTR (351 – 372)    5'   UUCACUA-UAUUCAU--UGAUCUGU 5’
                                  |||  : ||||:  |||||| :
miR-82                     3'      UGACCGAAAGUGCUACUAGA-GU 3’

daf-4 3’UTR (351 – 372)    5'     UUCACUAUAUUCAUUGAUCUGU 5’
                                     ||  :| |  ||||||| |
miR-1834                   3'   AACCUAGA-GUUACCAACUAGAGA 3’
```

```
daf-1 3'UTR (98 – 119)      5'  CACCCCCAUAUC-G-AUGAUCUCA 3'
                                 |   || || | | |:|||||||
miR-58                      3'   UAACGGCAU-GACUUGCUAGAGU 5'

daf-1 3'UTR (98 – 119)      5'   C-ACCCCCAUAUC-GAUGAUCUCA 3'
                                 |  |   || | | :|||||||||
miR-80                      3'  AGCCGAAAGU-U-GAUUACUAGAGU 5'

daf-1 3'UTR (98 – 119)      5'  CACCCCCA--UAU--CGAUGAUCUCA 3'
                                 |   | |  | |  |||||||||||
miR-81                      3'   U---GAUCGAAAGUGCUACUAGAGU 5'

daf-1 3'UTR (98 – 119)      5'  CACCCCCAUAU--CGAUGAUCUCA 3'
                                 ||   | | |  |||||||||||
miR-82                      3'   UGACCG-AAAGUGCUACUAGAGU 5'

daf-1 3'UTR (98 – 119)      5'     CACCCCCAUAUCGAUGAUCUCA 3'
                                    |  | || || | |||||||
miR-1834                    3'  AACCU-AGAGU-UACCAACUAGAGA 5'


daf-1 3'UTR (325 – 348)     5'  UUUUUUUUCUGUACCAUGAUUUCA 3'
                                   || :: |::   |:|||:|||
miR-58                      3'    UAACGGCAUGACUUGCUAGAGU 5'

daf-1 3'UTR (325 – 348)     5'  UUUUUUUUCUGUACCAUGAUUUCA 3'
                                |:  :|||| ::   |||||:|||
miR-80                      3'  AGCCGAAAGUUG-AUUACUAGAGU 5'

daf-1 3'UTR (325 – 348)     5'  UUUUUUUUCUGUACCAUGAUUUCA 3'
                                   :|  : | :|| |||||:|||
miR-81                      3'    UGAUCGAAAGUGCUACUAGAGU 5'

daf-1 3'UTR (325 – 348)     5'  UUUUUUUUCUGUACCAUGAUUUCA 3'
                                   :|  : | :|| |||||:|||
miR-82                      3'    UGACCGAAAGUGCUACUAGAGU 5'

daf-1 3'UTR (325 – 348)     5'   UUUUUUUUCUGUACCAUGAUUUCA 3'
                                 ||   |:|| :|    ||||:||
miR-1834                    3'   AACCUAGAGUUA-CCAACUAGAGA 5'
```

```
daf-7 3’UTR (54 – 77)      5’  AAUGAAUCAAAAUU-CAUGAUUUCA 3’
                                     :|   |:|  |:|||:|||
miR-58                     3'    UAACGG-CAUGACUUGCUAGAGU 5’

daf-7 3’UTR (54 – 77)      5’  AAUGAAUCAAAAUUCAUGAUUUCA 3’
                                 :|  |   ||:| |||||:|||
miR-80                     3'   AGCCGAAAGUUGAUUACUAGAGU 5’

daf-7 3’UTR (54 – 77)      5’  AAUGAAUCAAAAUUCAUGAUUUCA 3’
                                |:  | |     : |||||:|||
miR-81                     3'   UGA-UCGAAAGUGCUACUAGAGU 5’

daf-7 3’UTR (54 – 77)      5’  AAUGAAUCAAAAUUCAUGAUUUCA 3’
                                |:    |     : |||||:|||
miR-82                     3'   UGA-CCGAAAGUGCUACUAGAGU 5’

daf-7 3’UTR (54 – 77)      5’  AAUGAAUCAAAAUUCAUGAUUUCA 3’
                                 || |||  |||   ||||:||
miR-1834                   3'   AACCUAGAGUUACCAACUAGAGA 5’


daf-7 3’UTR (148 - 171)    5’  GGAGCUUCUCUUCUG-UCGGUCUCC 3’
                                  : | |: | |||  |||||||
miR-58                     3'     UAACGGCAUGACUUGCUAGAGU 5’

daf-7 3’UTR (148 - 171)    5’  GGAGCUUCUCUUCUGUCGGUCUCC 3’
                                  | :| ||  ||:  |:||||
miR-80                     3'   AGCCGAAAGUUGAUUACUAGAGU 5’

daf-7 3’UTR (148 - 171)    5’  GGAGCU-UCUCUUCUGUCGGUCUCC 3’
                                  :||  || |: :|  |:||||
miR-81                     3'     UGAUCGAAAGUGCUACUAGAGU 5’

daf-7 3’UTR (148 - 171)    5’  GGAGCU-UCUCUUCUGUCGGUCUCC 3’
                                  :||  || |: :|  |:||||
miR-82                     3'     UGACCGAAAGUGCUACUAGAGU 5’

daf-7 3’UTR (148 - 171)    5’  GGAGCUUCUCUUCUGUCGGUCUCC 3’
                                  |  ||||    || |:||||
miR-1834                   3'   AACCUAGAGUUACCAACUAGAGA 5’

daf-7 3’UTR (165 - 186)    5’  GGUCU-CCUUUCCCU---CGAUCUCA 3’
                                :| | ||  |  ||   ||||||||
miR-58                     3'   UA-ACGG-CA-UGACUUGCUAGAGU 5’
```

```
daf-7 3’UTR (165 - 186)    5'  GGUCUCCUUUC-CCU--CGAUCUCA 3’
                                 ||  |||||  ||   |||||||
miR-80                     3'    AGCCGAAAGUUGAUUACUAGAGU 5’

daf-7 3’UTR (165 - 186)    5'  GGUCU-CCUUUC-CCUCGAUCUCA 3’
                                : ||  ||||| |   |||||||
miR-81                     3'   U-GAUCGAAAGUGCUACUAGAGU 5’

daf-7 3’UTR (165 - 186)    5'  GGUCU-CCUUUC-CCUCGAUCUCA 3’
                                : ||  ||||| |   |||||||
miR-82                     3'   U-GACCGAAAGUGCUACUAGAGU 5’

daf-7 3’UTR (165 - 186)    5'    GGUCUCCUUUCCCUCGAUCUCA 3’
                                 ||  || :     | ||||||
miR-1834                   3'  AACC-UAGAGUUACCAACUAGAGA 5’
```

Table S6. Reduced IIS does not induce dauer formation in *mir-58f(-)*

	20ºC		25ºC	
**Genotype**	**% dauers**	**n**	**% dauers**	**n**
N2	0	1511	0	1107
mir-58f(-)	0	851	0	511
daf-2(e1370)	0.14	1442	100	1001
mir-58f(-);daf-2(e1370)	0(2.14[a])	748	0(100[a])	202

[a] % of arrested larvae that are not true dauers because they are able to pump and alae is not present.
